# Genetic glucocorticoid receptor variants differ between ethnic groups but do not explain variation in age of diabetes onset, metabolic and inflammation parameters in patients with type 2 diabetes

**DOI:** 10.3389/fendo.2023.1200183

**Published:** 2023-09-04

**Authors:** Mohamed Ahdi, Maaike C. Gerards, Paul H.M. Smits, Eelco W. Meesters, Dees P. M. Brandjes, Max Nieuwdorp, Victor E. A. Gerdes

**Affiliations:** ^1^ Department of Vascular Medicine, Amsterdam University Medical Centers (UMCs), Amsterdam, Netherlands; ^2^ Department of Molecular Biology, Atalmedial, Amsterdam, Netherlands; ^3^ Department of Internal Medicine, Spaarne Hospital, Hoofddorp, Netherlands

**Keywords:** glucocorticoid receptor, diabetes, glucocorticoid medication, cortisol, glucose metabolism, ethnicity, inflammation (markers)

## Abstract

**Aims:**

The effect of excess glucocorticoid receptor (GR) stimulation through glucocorticoid medication or cortisol on glucose metabolism is well established. There are genetic GR variants that result in increased or decreased GR stimulation. We aimed to determine the prevalence of genetic GR variants in different ethnic groups in a cohort of patients with type 2 diabetes, and we aimed to determine their association with age of diabetes onset and metabolic and inflammation parameters.

**Methods:**

A cross-sectional analysis was performed in a multiethnic cohort (n = 602) of patients with established type 2 diabetes. Polymorphisms in the GR gene that have previously been associated with altered glucocorticoid sensitivity (*TthIII*I, ER22/23EK N363S, *Bcl*I and 9β) were determined and combined into 6 haplotypes. Associations with age of diabetes onset, HbA1c, hs-CRP and lipid values were evaluated in multivariate regression models.

**Results:**

The prevalence of the SNPs of N363S and *Bcl*I was higher in Dutch than in non-Dutch patients. We observed a lower prevalence of the SNP 9β in Dutch, South(East) Asian and Black African patients versus Turkish and Moroccan patients. We did not detect an association between SNPs and diabetes age of onset or metabolic parameters. We only found a trend for lower age of onset and higher HbA1c in patients with 1 or 2 copies of haplotype 3 (*TthIII*I + 9β).

**Conclusions:**

The prevalence of genetic GR variants differs between patients of different ethnic origins. We did not find a clear association between genetic GR variants and age of diabetes onset or metabolic and inflammation parameters. This indicates that the clinical relevance of GR variants in patients with established type 2 diabetes is limited.

## Introduction

The onset and course of type 2 diabetes mellitus is determined by a combination of environmental and genetic risk factors. The effect of excess glucocorticoid receptor (GR) stimulation through glucocorticoid medication or cortisol on the incidence of type 2 diabetes is well established (1, 2). It is unknown whether genetic GR variants that are associated with increased GR stimulation also contribute to a diabetogenic phenotype. Ethnic origin is one of the factors associated with the incidence and course of type 2 diabetes and metabolic syndrome (3). Ethnicity as a determinant for disease consists of shared origin and genetics but also shared social and environmental background (4). If the prevalence of genetic GR variants differs in populations from different ethnicities and geographical regions, differences in functioning of this receptor could partly explain differences in onset and outcome of type 2 diabetes among ethnic groups.

The GR is expressed in almost every cell in the body (5). Binding of the GR by cortisol or glucocorticoid medication results in transrepression and transactivation of certain genes. Transrepression contributes to suppression of inflammation, and transactivation contributes to regulation of energy metabolism. Excess transactivation has effects that are comparable to metabolic derangements in type 2 diabetes (6). In clinical practice, we frequently encounter the effects of excess transactivation due to supraphysiological GR stimulation. Examples are acute disturbance of glucose metabolism due to high-dose glucocorticoid therapy and increased incidence of type 2 diabetes in Cushing syndrome (1, 2). Glucocorticoid signalling can also affect lipid metabolism, resulting in higher levels of triglycerides and total cholesterol (7).

The gene that encodes the glucocorticoid receptor (NR3C1) consists of 157,582 base pairs and is located on chromosome 5 (8). Single nucleotide polymorphisms (SNPs) can induce changes in the configuration and sensitivity of the GR, which may impact the binding and regulation of gene expression with glucocorticoids, and may subsequently affect inflammatory suppression and glucose metabolism (9). There are functional GR variants (SNPs) that can potentially change the transactivation and/or transrepression capacity of the GR gene. A schematic overview of the GR gene including the locations of these SNPs within the gene has been published before (10, 11).

GR variants B*cl*I (rs41423247) and N363S (rs6195) are associated with increased transactivation (sensitivity to glucocorticoids), whereas an SNP at ER22/23EK (rs6189) is associated with diminished transactivation. On the other hand, the GR variant 9β (rs6198) is associated with lower transrepression (10, 12). A fifth SNP, *TthIII*I (rs10052957) does not affect glucocorticoid sensitivity on itself but can result in glucocorticoid resistance in the presence of ER22/23EK (10). Form a clinical perspective, it has been observed that both BclI and N363S variations are linked to abdominal obesity, although there have been conflicting findings regarding N363S. Furthermore, N363S has been associated with higher levels of LDL-cholesterol and an increased risk of cardiovascular disease, while the ER22/23EK polymorphism has been associated with a reduced risk of dementia but an increased risk of major depression. Additionally, the 9β variant has been linked to increased inflammatory markers, rheumatoid arthritis, post-traumatic stress disorder, and cardiovascular disease (10, 13).The prevalence of the SNPs varies in previous studies, with a minor allele frequency (MAF) of 1.9-3% for ER22/23EK and 29.6-38.6% for B*cl*I (11, 14–17).

In this study, we aim to determine the association between genetic GR variants and the incidence and course of type 2 diabetes and metabolic syndrome in patients with established type 2 diabetes from different ethnic groups. We hypothesize that GR SNPs resulting in increased transactivation are associated with a lower age of diabetes onset and impaired glycemic control, and SNPs resulting in diminished transrepression are associated with a higher level of inflammation in patients with established type 2 diabetes. The second aim is to evaluate whether the prevalence of genetic GR variants differs between ethnic groups.

## Methods

We performed a cross-sectional analysis in a multi-ethnic cohort of patients with type 2 diabetes who were treated in secondary care (18). The participants consisted of consecutive individuals who visited the outpatient clinic of MC Slotervaart in Amsterdam for their annual comprehensive diabetes assessment between May 2009 and December 2010. Only those who provided written informed consent, and for whom DNA material was available for analysis were included in this study. For all participants, data on ethnic origin, diabetes onset, glucose- and lipid-lowering treatment, established complications, vital, anthropometric and laboratory parameters were registered. The study protocol was approved by the institutional review board.

The diagnosis of type 2 diabetes was based on the general practitioner (GP) referral letter in combination with clinical and biochemical characteristics determined at our clinic. GAD antibodies and C-peptide were determined in case of doubt regarding the type of diabetes. Age of diabetes onset was retrieved from the GP referral letter and checked with the patient. In case of discrepancy between referral letter and patient history, the age of onset as told by the patient was considered true. Ethnicity was determined according to the country of birth of either the patient or his or her parent and by last name analysis (4). The following ethnic groups were considered: native Dutch, Turkish, Moroccan, Southeast Asians (comprising 57% Hindustani and 25% Indonesians), and Black Africans (with 78% being Surinamese Creoles). Additional information can be found in the caption of **Table S1**.

### Laboratory assays

Blood samples were obtained by standard phlebotomy after a 10-hour overnight fast.

Depending on the patients’ informed consent form, an additional 10 ml EDTA-anticoagulated whole blood sample was collected. Following immediate centrifugation (15 minutes, 3000rpm, 1860g at 15°C), the isolated “buffy-coat” was carefully separated using a Pasteur pipette tube and stored in 0.5 ml vials at -70°C until assayed.

Total genomic DNA was isolated from the frozen ‘buffy-coat’, using the total nucleic acid (TNA) protocol on the MagNAPure LC (Roche Diagnostics). PCR primers (forwards and reverse) as well as MGB probes were designed using Primer Express Software v3.0.1 of Life Technologies. Five NR3C1 SNPs were determined by real-time polymerase chain reaction (RT-PCR): *TthIII*I (rs10052957: guanine > adenine), ER22/23EK (rs6189: guanine > adenine and rs6190: guanine > adenine), N363S (rs6195: adenine > guanine), B*cl*I (rs41423247: cytosine > guanine) and 9β (rs6198: adenine > guanine) followed by the allelic discrimination protocol on an ABI 7500 real-time PCR thermocycler (Thermo Fisher) as described previously (19). These SNPs combine into 6 haplotypes, as previously shown (11). For each haplotype, 3 genotype combinations were distinguished as carrying 0, 1, or 2 copies of the haplotype allele. To show that the designed assays were able to detect the indicated SNPs, all assays were validated before using well-characterized DNA kindly provided by P. Noordijk (Leiden University Medical Centre) from each individual SNP.

The routine analysis of these samples for HbA1c was performed using a Menarini (Adams™ HA-8160, Arkray Inc, Kyoto, Japan) automated HPLC analyser. Serum total- and HDL-cholesterol and triglycerides were determined using standard laboratory procedures within 4 hours after sampling with an automated analyser (Synchron^®^ LX20, Beckman Coulter Inc, Fullerton CA, USA). LDL-cholesterol was calculated using the Friedewald formula (20). High-sensitivity C-reactive protein (hs-CRP) was determined with a near infrared particle immunoassay rate methodology (Beckman Brea, CA).

### Statistical analysis

We estimated beta-coefficients for the change in age of diabetes onset, glycemic control, inflammation and lipid parameters for each SNP in linear regression models. Age, sex, diabetes duration, BMI, glucose and lipid-lowering medication and ethnicity were assessed as confounders if applicable. Potential confounders were selected if we presumed a theoretical relationship with SNP status and outcome, in combination with a statistical association (21). Outcome variables that had a non-normal distribution were transformed to approximate normality. To ease interpretation, we presented back-transformed values of those variables in the outcome tables.

We performed an *a priori* power estimation on the difference in age of diabetes onset in the absence or presence of different polymorphisms. The power generally increases as a SNP is more prevalent (and as the effect on glucocorticoid sensitivity is stronger) (22). For the least prevalent SNP - ER22/23EK (94% wild type) - univariate regression analysis with a 5% significance level will have 85% power to detect the difference of 4 years (standard deviation ± 9) in age of onset between patients with a glucocorticoid-resistant genotype and patients with a glucocorticoid-sensitive genotype when the total sample size is 624 patients.

## Results

### Patients and genotyping

From a total of 983 patients with type 2 diabetes, 602 patients had available DNA samples and were included. There were no significant differences in demographics, clinical variables, and complications between patients with and patients without available DNA data (**Table S1**). Overall, patients had a reasonably well-regulated diabetes with an average HbA1c level of 7.3% (54 mmol/mol) and the average diabetes duration was 11.9 ± 8.5 years. Fourty-four percent of the participants were of non-native Dutch origin, comprising individuals from Turkish (n = 45), Moroccan (n = 101), Southeast Asian (n = 79), and black African (n = 40) backgrounds. Non-Dutch patients were as compared with Dutch patients, less frequently males (46 versus 59%, p < 0.001), were younger (mean 57.4 versus 65.8 years), their average age of diabetes onset was 8.9 years earlier, and they had a poorer level of glycemic control (mean 7.6 versus 7.0% [60 vs. 53 mmol/mol]. The characteristics of the study population are shown in **Table S1**.

### SNPs of the glucocorticoid receptor gene

In 557 patients (93%), at least 1 SNP could be determined. As shown in **Table 1**, the minor allele frequency varied from 1.6% (ER22/23EK) to 31.6% (*Bcl*I). The prevalence of SNPs was not associated with sex or age. We found a higher prevalence of the N363S SNP and a lower prevalence of the *Bcl*I CC genotype in Dutch than in non-Dutch patients (p < 0.01). Additionally, we observed a difference in the prevalence of 9β in Dutch, Southeast Asian and Black African patients versus Turkish and Moroccan patients, but this difference was not significant (p = 0.094).

**Table 1:** Prevalence of SNPs of the glucocorticoid receptor gene by ethnic origin.

Genotype		Dutch *n (%)*	Turkish *n (%)*	Moroccan *n (%)*	SE Asian *n (%)*	Black African *n (%)*	P^*^
**TTH111I (rs10052957)**	CC	162 (49.7)	24 (54.5)	41 (42.3)	52 (67.5)	18 (46.2)	
	CT	139 (42.6)	18 (40.9)	46 (47.4)	24 (31.2)	19 (48.7)	
	TT	25 (7.7)	2 (4.5)	10 (10.3)	1 (1.3)	2 (5.1)	0.211
**ER22/23EK (rs6189/rs6190)**	GG/GG	319 (95.8)	43 (95.6)	98 (100)	77 (98.7)	38 (95)	
	GA/GA	14 (4.2)	2 (4.4)	0 (0)	1 (1.3)	2 (5)	0.211
**N363S (rs6195)**	AA	298 (90.3)	44 (97.8)	96 (97.0)	76 (97.4)	39 (100)	
	AG	30 (9.1)	1 (2.2)	3 (3.0)	2 (2.6)	0 (0)	
	GG	2 (0.6)	0 (0)	0 (0)	0 (0)	0 (0)	<0.001
***BCL*I *(rs41423247)***	CC	123 (38.9)	23 (57.5)	46 (48.9)	42 (54.5)	25 (67.6)	
	CG	163 (51.6)	16 (40)	35 (37.2)	31 (40.3)	9 (24.3)	
	GG	30 (9.5)	1 (2.5)	13 (13.8)	4 (5.2)	3 (8.1)	0.002
**9β (rs6198)**	AA	232 (71.4)	25 (56.8)	56 (58.9)	64 (83.1)	34 (87.2)	
	AG	93 (28.6)	19 (43.2)	39 (41.1)	13 (16.9)	5 (12.8)	0.094

*Statistical significance of differences between ethnic groups is tested through a chi square test for trend.

### Association SNPs of the GR gene with age of diabetes onset and parameters of metabolic syndrome

In the overall study population, diabetes was diagnosed at the age of 50.4 years. We could not detect a clear influence of the SNPs of the GR gene and age of onset, except for patients who were heterozygous for the 9β SNP (50.9 versus 49.2, p adj 0.02). Patients with the 9β SNP showed a trend toward higher HbA1c and CRP (**Table 2**). Patients with at least 1 copy of the N363S polymorphism had a lower LDL cholesterol. We did not observe any effect of the *TthIII*I, ER22/23EK and *Bcl*I polymorphisms on glycemic control, inflammation or lipid parameters.

**Table 2:** Association of SNPs of the glucocorticoid receptor gene with clinical characteristics.

Genotype		N	MAF *(%)*	Age DM onset *(years)*	HbA1c *(%)*	hs-CRP *(mmol/l)*	Total chol *(mmol/l)*	Triglyc *(mmol/l)*	LDL chol *(mmol/l)*
**TTH111I (rs10052957)**	**CC**	297		50.5 (11.4)	7.2 (1.2)	4.4 (6.6)	4.2 (1.0)	1.7 (1.0)	2.3 (0.8)
	**T vs. CC**	286	28.0	50.3 (11.6)	7.4 (1.3)	4.6 (6.8)	4.3 (1.0)	1.9 (2.3)	2.4 (0.8)
adjusted	Beta			-0.52 (0.91)	0.15 (0.10)	0.08 (0.55)	0.03 (0.08)	0.16 (0.15)	-0.02 (0.06)
	P			0.57	0.12	0.88	0.68	0.26	0.75
**ER22/23EK (rs6189/rs6190)**	**GG/GG**	575		50.5 (11.5)	7.3 (1.3)	4.5 (6.7)	4.2 (1.0)	1.8 (1.8)	2.4 (0.8)
	**A vs GG/GG**	19	1.6	51.4 (10.7)	7.2 (0.6)	3.6 (3.2)	4.0 (0.8)	1.6 (0.9)	2.2 (0.8)
adjusted	Beta			-0.12 (2.54)	-0.25 (0.27)	-0.62 (1.53)	-0.22 (0.23)	-0.24 (0.41)	-0.21 (0.18)
	P			0.96	0.36	0.69	0.33	0.57	0.24
**N363S (rs6195)**	**AA**	553		50.3 (11.5)	7.3 (1.3)	4.5 (6.7)	4.2 (1.0)	1.7 (1.0)	2.4 (0.8)
	**G vs AA**	38	3.4	52.7 (11.3)	7.2 (1.0)	3.9 (6.8)	4.2 (0.6)	1.9 (1.0)	2.1 (0.5)
adjusted	Beta			0.29 (1.86)	-0.01 (0.20)	-0.81 (1.11)	-0.11 (0.16)	0.17 (0.17)	-0.24 (0.13)
	P			0.87	0.95	0.46	0.51	0.33	0.06
***BCL*I *(rs41423247)***	**CC**	259		50.0 (11.3)	7.3 (1.2)	4.1 (5.7)	4.2 (0.9)	1.7 (0.9)	2.4 (0.8)
	**G vs. CC**	305	31.6	50.8 (11.7)	7.2 (1.3)	4.7 (7.1)	4.2 (1.1)	1.9 (2.3)	2.3 (0.8)
adjusted	Beta			-0.51 (0.94)	0.03 (0.10)	0.55 (0.54)	-0.05 (0.08)	0.18 (0.15)	-0.09 (0.06)
	P			0.59	0.75	0.31	0.54	0.23	0.18
**9β (rs6198)**	**AA**	411		50.9 (11.4)	7.2 (1.2)	4.4 (6.5)	4.2 (1.0)	1.8 (2.0)	2.3 (0.8)
	**G vs AA**	169	14.6	49.2 (11.4)	7.4 (1.4)	4.9 (7.2)	4.3 (0.9)	1.8 (1.0)	2.4 (0.8)
adjusted	Beta			-2.25 (0.99	0.16 (0.11)	0.41(0.61)	0.01 (0.09)	0.02 (0.16)	-0.03 (0.07)
	P			0.02	0.14	0.50	0.90	0.90	0.64

Data are presented as mean (sd). Adjustments in the multivariate linear regression model: Age of onset was adjusted for sex and ethnicity; HbA1c was adjusted for sex, ethnicity, diabetes duration, insulin use and metformin use; hsCRP was adjusted for age and sex; lipid spectrum was adjusted for sex, age, use of lipid lowering medication and metformin.

### Association of haplotypes of the GR gene with age of diabetes onset and parameters of metabolic syndrome

Haplotype 1 (which does not contain any SNP, wild type) had a minor allele frequency of 47.9%. The minor allele frequency of other haplotypes varied from 1.6% (haplotype 6) to 20.1% (haplotype 2). Patients who had 1 or 2 copies of haplotype 3 showed a trend toward a lower age of diabetes onset and a higher HbA1c, which is in accordance with the results of the individual haplotypes (**Table 3**). Patients who had at least 1 copy of haplotype 5 showed a trend toward lower LDL cholesterol. No associations were found for the other haplotypes.

**Table 3:** Association of haplotypes of the GR receptor gene with clinical characteristics.

Haplotype	Copies	N	Age DM onset (years)	HbA1c (%)	Hs-CRP (mmol/l)	Total chol. (mmol/l)	Triglycer. (mmol/l)	LDL chol. (mmol/l)
**1**	0	147	49.5 (11.7)	7.3 (1.3)	4.7 (7.0)	4.2 (1.0)	1.8 (1.1)	2.3 (0.7)
**wild type**	1	288	51.0 (11.6)	7.3 (1.2)	4.5 (6.6)	4.2 (1.0)	1.7 (1.0)	2.4 (0.8)
	2	124	50.3 (11.2)	7.3 (1.2)	4.0 (6.0)	4.2 (0.9)	1.6 (1.0)	2.3 (0.8)
adjusted	Beta		1.53 (0.68	-0.08 (0.07)	-0.13 (0.40)	0.01 (0.06)	-0.11 (0.06)	0.05 (0.05)
	P		0.03	0.27	0.74	0.88	0.09	0.24
**2**	0	360	50.6 (11.4)	7.3 (1.2)	4.3 (6.3)	4.2 (1.0)	1.7 (1.0)	2.3 (0.8)
***BCL*I**	1	178	50.2 (11.8)	7.3 (1.3)	4.7 (7.3)	4.2 (1.0)	1.7 (1.0)	2.3 (0.8)
	2	24	50.5 (11.3)	7.3 (1.1)	4.0 (3.6)	4.3 (1.0)	1.9 (1.1)	2.4 (0.9)
adjusted	Beta		-0.80 (0.81)	0.02 (0.08)	0.14 (0.48)	0.00 (0.07)	0.04 (0.08)	-0.01 (0.06)
	P		0.32	0.78	0.76	0.97	0.58	0.84
**3**	0	431	50.9 (11.4)	7.3 (1.2)	4.4 (6.5)	4.2 (1.0)	1.8 (2.0)	2.3 (0.8)
***TthIII*I + 9β**	1	134	49.3 (11.3)	7.4 (1.4)	4.7 (7.3)	4.3 (1.0)	1.8 (1.1)	2.4 (0.8)
	2	12	44.5 (14.2)	7.8 (1.4)	5.1 (6.2)	3.8 (0.6)	1.7 (0.7)	2.1 (0.4)
adjusted	Beta		-2.33 (0.93)	0.14 (0.10)	0.14 (0.56)	-0.01 (0.08)	0.04 (0.15)	-0.03 (0.07)
	P		0.01	0.14	0.80	0.92	0.79	0.64
**4**	0	442	50.1 (11.5)	7.3 (1.2)	4.3 (6.3)	4.2 (1.0)	1.7 (1.0)	2.4 (0.8)
***TthIII*I + *BCL*I**	1	120	52.2 (11.6)	7.2 (1.3)	5.0 (7.3)	4.2 (1.0)	1.8 (1.2)	2.3 (0.7)
	2	120	45.0 (10.8)	7.4 (0.9)	2.0 (1.3)	4.7 (0.8)	1.9 (1.4)	2.7 (0.7)
adjusted	Beta		0.45 (1.05)	-0.01(0.11)	0.16 (0.61)	-0.02 (0.09)	0.04 (0.10)	-0.05 (0.07)
	P		0.67	0.93	0.79	0.83	0.67	0.49
**5**	0	554	50.3 (11.5)	7.3 (1.3)	4.5 (6.7)	4.2 (1.0)	1.7 (1.0)	2.4 (0.8)
**N363S**	1	38	52.9 (11.2)	7.3 (1.0)	3.8 (6.9)	4.1 (0.6)	1.9 (1.0)	2.1 (0.5)
	2	38	48.5 (17.7)	6.2 (0.3)	6.7 (6.7)	4.8 (0.9)	1.6 (0.8)	2.8 (1.0)
adjusted	Beta		0.00 (1.72)	-0.03(0.18)	-0.65 (1.03)	-0.07 (0.15)	0.14 (0.16)	-0.18 (0.12)
	P		1.00	0.87	0.53	0.65	0.38	0.13
**6**	0	577	50.4 (11.5)	7.3 (1.3)	4.5 (6.7)	4.2 (1.0)	1.8 (1.8)	2.4 (0.8)
***TthIII*I + ER22/ 23EK + 9β**	1	19	51.4 (10.7)	7.2 (0.6)	3.6 (3.2)	4.0 (0.8)	1.6 (0.9)	2.2 (0.8)
	2	0	–	–	–	–	–	–
adjusted	Beta		-0.09 (2.54)	-0.25 (0.27)	-0.62(1.53)	-0.22 (0.23)	-0.23 (0.41)	-0.21 (0.18)
	P		0.97	0.36	0.69	0.33	0.57	0.24

Data are presented as mean (sd). Adjustments in the multivariate linear regression model: Age of onset was adjusted for sex and ethnicity; HbA1c was adjusted for sex, ethnicity, diabetes duration, insulin use and metformin use; hsCRP was adjusted for age and sex; lipid spectrum was adjusted for sex, age, use of lipid lowering medication and metformin.

## Discussion

We studied the association between genetic variants of the GR and metabolic and inflammation parameters in a multiethnic cohort of patients with established type 2 diabetes in secondary care. We observed a different prevalence of genetic variants between patients of different ethnic origins. We did not find a clear association between genetic variants and age of diabetes onset, glycemic control, lipid parameters or inflammation, and we found only a trend for lower age of onset for patients with haplotype 3. This suggests that the clinical relevance of these genetic variants for the onset of diabetes and the course of established diabetes seems to be minor.

The development of type 2 diabetes is a combination of genetic and environmental risk factors. Whereas mutations underlying monogenic diabetes have direct clinical consequences, genetic variants in multifactorial forms of diabetes have a much weaker association (23). In patients with established diabetes, such as in our study population, HbA1c and lipid parameters are affected by medication and BMI. Despite adjusting for these confounding factors, we did not find an association. Additionally, for the time of diabetes onset – a parameter that is unbiased by glucose-lowering treatment - we did not find an association with genetic variants of the glucocorticoid receptor.

The SNPs N363S and ER22/23EK, which were previously associated with increased and decreased transactivation, respectively, did not affect the age of diabetes onset. Interestingly, the N363S SNP, which we hypothesized to result in diabetes onset at a younger age, showed a trend towards later diabetes onset. Despite the increased prevalence of N363S in Dutch patients compared to patients of Turkish and Moroccan origin, Dutch patients were diagnosed with diabetes at a later age. In patients with at least one copy of SNP 9β, we observed a trend for a higher level of hs-CRP, which is in line with our hypothesis.

Although specific effects on transrepression and transactivation have been established *in vitro* for all analysed SNPs, clinical studies have shown contradictory results. For example, the ER22/23EK SNP reduced GC-induced transactivation *in vitro*, and supportive evidence was found by increased insulin sensitivity and lower fasting insulin concentration in a Dutch cohort (24). However, ER22/23EK was associated with higher HbA1c levels in a cohort of patients older than 85 years old (17). Minor allele frequency was not different between these cohorts, arguing against an age difference as an explanation for the contradictory findings. Glucose metabolism is a highly regulated process in which multiple genetic and environmental factors are intertwined with an eventual effect of glucocorticoid sensitivity (25). The absence of an association in our study suggests that there is no clinically relevant effect of GR variants on glucose metabolism and that the previous contradictory findings may have arisen by chance.

Our study has both strengths and weaknesses. A strength of our study is the extensive data with both detailed information on treatment as well as laboratory parameters and therefore the ability to correct for possible confounders. By including all consecutive patients in our clinic, we established a cohort that is representative for the secondary care diabetes population in an urban area. However, the heterogeneity of our study population regarding age, diabetes duration and origin might also have blunted the effect of genetic variants on metabolic parameters. A weakness of our study arises from the cross-sectional nature of the cohort. The age of diabetes onset is determined retrospectively, and we cannot exclude the possibility of recall or information bias on this outcome parameter. Although a diagnostic delay in type 2 diabetes is frequently observed, in previous studies, the duration of delay was not affected by ethnicity of the patient (26, 27). Furthermore, we do not have data on the socioeconomic position of patients, which could be an uncontrolled confounder between ethnicity and diabetes outcome parameters.

In conclusion, we observed that the prevalence of SNPs of the glucocorticoid receptor was different between ethnic groups. We found a modest association between the 9β SNP of the GR and the level of systemic inflammation in patients with established and well-regulated type 2 diabetes. However, genetic variants of the GR did not explain the variation in age of diabetes onset and level of glycemic control; therefore, its clinical relevance for patients with established type 2 diabetes is limited.

## Data availability statement

The datasets presented in this study can be found in online repositories. The names of the repositories and accession numbers are as follows: ER22/23EK (rs6189): VCV000155925.10 - ClinVar - NCBI (nih.gov); 9β (rs6198): VCV000351314.5 - ClinVar - NCBI (nih.gov); N363S (rs6195 has merged into rs56149945): VCV000016150.9 - ClinVar - NCBI (nih.gov); TTH111I (rs10052957): rs10052957 RefSNP Report - dbSNP - NCBI (nih.gov); BCLI (rs41423247): rs41423247 RefSNP Report - dbSNP - NCBI (nih.gov).

## Ethics statement

All procedures followed were in accordance with the ethical standards of the responsible committee on human experimentation(institutional and national) and with the Helsinki Declaration of 1975, as revised in 2008 (5).

## Author contributions

MA and MG (shared first authors) designed the study protocol, wrote the manuscript, collected data, and performed statistical analyses. PS performed the molecular biological procedures contributed to the discussion, and reviewed/edited the manuscript. VG and DB designed the study protocol contributed to the discussion, and reviewed/edited the manuscript. EM and MN contributed to the discussion and reviewed/edited the manuscript. MA and MG had full access to all data in the study and take responsibility for the integrity of data and the accuracy of data analysis. All authors contributed to the article and approved the submitted version.
