# Supplementary material for: Genetic glucocorticoid receptor variants differ between ethnic groups but do not explain variation in age of diabetes onset, metabolic and inflammation parameters in patients with type 2 diabetes
Source: Front Endocrinol (Lausanne). 2023 Sep 4;14:1200183. doi: 10.3389/fendo.2023.1200183 (PMC10507347; doi:10.3389/fendo.2023.1200183)
Supplement: Supplementary file 1 [file Table_1.docx]

**Supplementary Table S1 Population characteristics by availability of DNA samples**

| **Characteristic ^^^** | **All patients** ^*^ **N= 983** | **Without GCR data** **N= 381** | **With GCR data**  **N= 602** | **P** |
| --- | --- | --- | --- | --- |
| **Demographics, median (IQR) ^a^ or n (%) ^b^** |  |  |  |  |
| Age (years) | 63 (53-71) | 64 (56-72) | 62 (55-70) | 0.059 |
| Diabetes duration (years) | 11 (6-16) | 11 (6-17) | 11 (6-16) | 0.186 |
| Male gender | 511 (52) | 195 (51) | 316 (53) | 0.698 |
| Never smoking | 455 (47) | 169 (45) | 268 (48) | 0.300 |
| **Ethnicity, n (%) ^b^** |  |  |  |  |
| Dutch | 504 (51) | 189 (50) | 315 (52) | 0.094 |
| Turkish | 81 (8) | 38 (10) | 43 (7) |  |
| Moroccan | 185 (19) | 83 (22) | 102 (17) |  |
| Hindustani | 61 (6) | 18 (5) | 43 (7) |  |
| Black African | 56 (6) | 22 (6) | 34 (6) |  |
| Others | 96 (10) | 31 (8) | 65 (11) |  |
| **Complications, n (%) ^b^** |  |  |  |  |
| Nephropathy | 497 (51) | 184 (49) | 313 (53) | 0.265 |
| Retinopathy | 265 (27) | 102 (27) | 163 (27) | 0.997 |
| Neuropathy | 236 (24) | 85 (23) | 151 (25) | 0.340 |
| Macrovascular disease | 304 (31) | 128 (34) | 176 (29) | 0.156 |
| **Measurements – median (IQR) ^a^** |  |  |  |  |
| Body mass index (kg/m2) | 30.5 (27.5-34.6) | 30.4 (27.6-34.8) | 30.5 (27.5-34.4) | 0.999 |
| Systolic blood pressure (mmHg) | 127 (116-138) | 126 (115-136) | 127 (116-140) | 0.032 |
| HbA1c (%) | 7.1 (6.4-7.9) | 7.2 (6.6-8.1) | 7.1 (6.4-7.8) | 0.036 |
| HbA1c (mmol/mol) | 54 (46-63) | 55 (49-65) | 54 (46-62) |  |
| Total cholesterol (mmol/L) | 4.1 (3.6-4.8) | 4.1 (3.6-4.8) | 4.1 (3.5-4.8) | 0.885 |
| HDL cholesterol (mmol/L) | 1.0 (0.9-1.3) | 1.0 (0.9-1.2) | 1.0 (0.9-1.3) | 0.778 |
| Triglycerides (mmol/L) | 1.5 (1.1-2.2) | 1.4 (1.0-2.0) | 1.5 (1.1-2.2) | 0.232 |
| LDL-cholesterol (mmol/L) | 2.3 (1.8-2.8) | 2.3 (1.9-2.8) | 2.2 (1.8-2.8) | 0.390 |
| Hs-CRP (mmol/l) | 2.5 (1.1-5.2) | 2.4 (1.1-5.1) | 2.6 (1.1-5.2) | 0.509 |
| **Therapy, n (%) ^b^** |  |  |  |  |
| Metformin | 868 (89) | 339 (89) | 529 (88) | 0.617 |
| Insulin | 666 (68) | 247 (65) | 419 (70) | 0.119 |

Details on data collection and measurements of this cohort are described elsewhere (18).
^a^ Mann-Whitney U test and ^b^ Chi Square test for patients without and patients with available DNA data.
* Corresponding means (sd) for the overall cohort (n=983): Age, 62.7 (11.6) years; diabetes duration 12.0 (8.2) years; BMI 31.3 (5.8) kg/m2; Systolic BP 128 (17) mmHg; HbA1c 56 (13) mmol/mol; non-HDL cholesterol 3.2 (1.0) mmol/l; hs-CRP 5.2 (20.6) mmol/l.
^ Missing data for patients without GCR data (•) and patients with GCR data (+): never smoking (• n=2; + n=6), nephropathy (• n=5; + n=7), retinopathy (• n=6; + n=3), neuropathy (• n=3; + n=2), macrovascular disease (• n=1; + n=3), systolic BP (• n=21; + n=26), BMI (• n=9; + n=16), HbA1c (• n=2), non-HDLc (• n=2; + n=1), hs-CRP (• n=7; + n=6), metformin (• n=1; + n=2).
