# Supplementary material for: Genetic glucocorticoid receptor variants differ between ethnic groups but do not explain variation in age of diabetes onset, metabolic and inflammation parameters in patients with type 2 diabetes
Source: Front Endocrinol (Lausanne). 2023 Sep 4;14:1200183. doi: 10.3389/fendo.2023.1200183 (PMC10507347; doi:10.3389/fendo.2023.1200183)
Supplement: Supplementary file 2 [file DataSheet_1.pdf]

SUPPLEMENTARY DATA ONE WAY ANOVA TABLE 1

Oneway

Descriptives

		N	Mean	Std. Deviation	Std. Error	95% Confidence Interval for Mean		Minimum	Maximum
						Lower Bound	Upper Bound		
Age of subject	The Netherlands	336	65,81	10,579	,577	64,68	66,95	37	89
	Turkey	45	58,51	11,849	1,766	54,95	62,07	38	80
	Moroccan	101	57,01	10,513	1,046	54,93	59,09	25	79
	SE Asian	79	57,47	12,022	1,353	54,78	60,16	29	86
	Black African	40	57,20	9,587	1,516	54,13	60,27	37	79
	Total	601	62,12	11,552	,471	61,19	63,04	25	89
Age of diabetes onset	The Netherlands	336	54,3363	11,12306	,60681	53,1427	55,5300	28,00	81,00
	Turkey	45	45,2889	8,83336	1,31680	42,6351	47,9427	30,00	63,00
	Moroccan	101	44,6436	10,59866	1,05461	42,5513	46,7359	17,00	65,00
	SE Asian	79	45,9367	10,34203	1,16357	43,6202	48,2532	25,00	79,00
	Black African	40	46,6750	9,04798	1,43061	43,7813	49,5687	30,00	69,00
	Total	601	50,4160	11,51130	,46956	49,4938	51,3381	17,00	81,00
Diabetes duration	The Netherlands	336	11,476	7,5393	,4113	10,667	12,285	,0	51,0
	Turkey	45	13,222	7,2704	1,0838	11,038	15,406	,0	29,0
	Moroccan	101	12,366	6,3541	,6323	11,112	13,621	1,0	31,0
	SE Asian	79	11,532	7,3499	,8269	9,885	13,178	,0	36,0
	Black African	40	10,525	5,7244	,9051	8,694	12,356	,0	21,0
	Total	601	11,700	7,2019	,2938	11,124	12,277	,0	51,0
Body mass index (kg/m2)	The Netherlands	332	31,155	6,0498	,3320	30,502	31,809	14,8	52,7
	Turkey	44	32,806	4,8076	,7248	31,345	34,268	24,9	43,3
	Moroccan	101	31,842	4,7100	,4687	30,913	32,772	22,6	45,9
	SE Asian	79	29,337	4,8932	,5505	28,240	30,433	18,6	44,9
	Black African	39	32,145	6,1541	,9854	30,150	34,140	21,4	43,7
	Total	595	31,217	5,6703	,2325	30,761	31,674	14,8	52,7
hsCRP	The Netherlands	336	4,589	7,1811	,3918	3,819	5,360	,0	54,7
	Turkey	45	4,679	5,2841	,7877	3,092	6,267	,4	26,2
	Moroccan	101	4,736	6,1517	,6121	3,522	5,950	,3	51,0
	SE Asian	79	3,319	3,4063	,3832	2,556	4,082	,0	16,2
	Black African	40	5,083	8,8137	1,3936	2,264	7,901	,2	48,2
	Total	601	4,486	6,6278	,2704	3,955	5,017	,0	54,7
HbA1c (%)	The Netherlands	336	7,022	1,0360	,0565	6,911	7,133	5,2	12,3
	Turkey	45	8,120	1,4634	,2182	7,680	8,560	6,0	13,1
	Moroccan	101	7,719	1,4408	,1434	7,434	8,003	5,4	12,3
	SE Asian	79	7,372	1,1897	,1339	7,106	7,639	5,4	10,5
	Black African	40	7,485	1,5199	,2403	6,999	7,971	5,7	13,2
	Total	601	7,298	1,2501	,0510	7,198	7,398	5,2	13,2
Fasting glucose (mmol/L)	The Netherlands	336	8,291	2,5379	,1385	8,019	8,563	,0	18,0
	Turkey	45	8,638	3,2532	,4850	7,660	9,615	3,8	18,3
	Moroccan	101	8,942	2,7441	,2731	8,400	9,483	3,9	17,6
	SE Asian	79	8,220	2,4146	,2717	7,679	8,761	4,0	15,9
	Black African	40	7,858	3,2152	,5084	6,829	8,886	,0	18,2
	Total	601	8,388	2,6723	,1090	8,174	8,602	,0	18,3

Total cholesterol	The Netherlands	336	4,243	1,0189	,0556	4,134	4,352	1,9	9,5
	Turkey	45	4,117	,9153	,1364	3,842	4,392	2,6	7,4
	Moroccan	101	4,193	,9826	,0978	3,999	4,387	1,9	6,5
	SE Asian	79	4,090	,8883	,0999	3,891	4,289	2,2	7,7
	Black African	40	4,600	1,2812	,2026	4,190	5,009	1,9	7,9
	Total	601	4,229	1,0120	,0413	4,148	4,310	1,9	9,5
HDL cholesterol	The Netherlands	336	1,099	,3499	,0191	1,062	1,137	,3	3,1
	Turkey	45	,947	,2319	,0346	,877	1,016	,6	1,7
	Moroccan	101	1,043	,2912	,0290	,986	1,101	,5	1,9
	SE Asian	79	1,074	,3103	,0349	1,005	1,144	,5	2,0
	Black African	40	1,344	,4461	,0705	1,202	1,487	,5	2,2
	Total	601	1,092	,3438	,0140	1,064	1,119	,3	3,1
Triglycerides	The Netherlands	336	1,924	2,1332	,1164	1,695	2,153	,3	36,6
	Turkey	45	1,996	1,3417	,2000	1,593	2,399	,6	6,2
	Moroccan	101	1,580	,9019	,0897	1,401	1,758	,6	4,5
	SE Asian	79	1,596	,9793	,1102	1,376	1,815	,5	6,7
	Black African	40	1,352	1,2364	,1955	,956	1,747	,4	7,9
	Total	601	1,790	1,7524	,0715	1,650	1,931	,3	36,6
LDL cholesterol	The Netherlands	334	2,302	,7828	,0428	2,218	2,387	,8	5,1
	Turkey	44	2,232	,6837	,1031	2,024	2,440	,6	3,7
	Moroccan	101	2,432	,8040	,0800	2,273	2,590	,4	4,3
	SE Asian	79	2,299	,7243	,0815	2,137	2,462	1,2	5,6
	Black African	39	2,725	1,0685	,1711	2,378	3,071	,8	5,8
	Total	597	2,346	,7993	,0327	2,282	2,410	,4	5,8

ANOVA

		Sum of Squares	df	Mean Square	F
Age of subject	Between Groups	10482,354	4	2620,588	22,447
	Within Groups	69581,493	596	116,747	
	Total	80063,847	600		
Age of diabetes onset	Between Groups	11857,138	4	2964,285	26,116
	Within Groups	67648,868	596	113,505	
	Total	79506,007	600		
Diabetes duration	Between Groups	223,411	4	55,853	1,077
	Within Groups	30896,679	596	51,840	
	Total	31120,090	600		
Body mass index (kg/m2)	Between Groups	464,838	4	116,209	3,680
	Within Groups	18633,774	590	31,583	
	Total	19098,612	594		
hsCRP	Between Groups	133,460	4	33,365	,758
	Within Groups	26222,956	596	43,998	
	Total	26356,416	600		
HbA1c (%)	Between Groups	75,770	4	18,943	13,099
	Within Groups	861,847	596	1,446	
	Total	937,618	600		
Fasting glucose (mmol/L)	Between Groups	50,396	4	12,599	1,773
	Within Groups	4234,270	596	7,104	
	Total	4284,666	600		

Total cholesterol	Between Groups	7,792	4	1,948	1,914
	Within Groups	606,737	596	1,018	
	Total	614,529	600		
HDL cholesterol	Between Groups	3,777	4	,944	8,385
	Within Groups	67,127	596	,113	
	Total	70,905	600		
Triglycerides	Between Groups	23,091	4	5,773	1,891
	Within Groups	1819,476	596	3,053	
	Total	1842,567	600		
LDL cholesterol	Between Groups	7,710	4	1,928	3,059
	Within Groups	373,094	592	,630	
	Total	380,804	596		

ANOVA

		Sig.
Age of subject	Between Groups	<,001
	Within Groups	
	Total	
Age of diabetes onset	Between Groups	<,001
	Within Groups	
	Total	
Diabetes duration	Between Groups	,367
	Within Groups	
	Total	
Body mass index (kg/m2)	Between Groups	,006
	Within Groups	
	Total	
hsCRP	Between Groups	,553
	Within Groups	
	Total	
HbA1c (%)	Between Groups	<,001
	Within Groups	
	Total	
Fasting glucose (mmol/L)	Between Groups	,133
	Within Groups	
	Total	
Total cholesterol	Between Groups	,107
	Within Groups	
	Total	
HDL cholesterol	Between Groups	<,001
	Within Groups	
	Total	

Triglycerides	Between Groups	,110
	Within Groups	
	Total	
LDL cholesterol	Between Groups	,016
	Within Groups	
	Total	

ANOVA Effect Sizes[a,b]

		Point Estimate	95% Confidence Interval Lower	Upper
Age of subject	Eta-squared	,131	,081	,177
	Epsilon-squared	,125	,074	,171
	Omega-squared Fixed-effect	,125	,074	,171
	Omega-squared Random-effect	,034	,020	,049
Age of diabetes onset	Eta-squared	,149	,096	,197
	Epsilon-squared	,143	,090	,191
	Omega-squared Fixed-effect	,143	,090	,191
	Omega-squared Random-effect	,040	,024	,056
Diabetes duration	Eta-squared	,007	,000	,020
	Epsilon-squared	,001	-,007	,013
	Omega-squared Fixed-effect	,001	-,007	,013
	Omega-squared Random-effect	,000	-,002	,003
Body mass index (kg/m2)	Eta-squared	,024	,002	,048
	Epsilon-squared	,018	-,004	,042
	Omega-squared Fixed-effect	,018	-,004	,042
	Omega-squared Random-effect	,004	-,001	,011
hsCRP	Eta-squared	,005	,000	,015
	Epsilon-squared	-,002	-,007	,008
	Omega-squared Fixed-effect	-,002	-,007	,008
	Omega-squared Random-effect	,000	-,002	,002
HbA1c (%)	Eta-squared	,081	,040	,120
	Epsilon-squared	,075	,033	,114
	Omega-squared Fixed-effect	,075	,033	,114
	Omega-squared Random-effect	,020	,009	,031
Fasting glucose (mmol/L)	Eta-squared	,012	,000	,028
	Epsilon-squared	,005	-,007	,022
	Omega-squared Fixed-effect	,005	-,007	,022
	Omega-squared Random-effect	,001	-,002	,005
Total cholesterol	Eta-squared	,013	,000	,030
	Epsilon-squared	,006	-,007	,023
	Omega-squared Fixed-effect	,006	-,007	,023
	Omega-squared Random-effect	,002	-,002	,006
HDL cholesterol	Eta-squared	,053	,020	,087
	Epsilon-squared	,047	,013	,081
	Omega-squared Fixed-effect	,047	,013	,080
	Omega-squared Random-effect	,012	,003	,021
Triglycerides	Eta-squared	,013	,000	,030
	Epsilon-squared	,006	-,007	,023
	Omega-squared Fixed-effect	,006	-,007	,023
	Omega-squared Random-effect	,001	-,002	,006
LDL cholesterol	Eta-squared	,020	,001	,042
	Epsilon-squared	,014	-,006	,035
	Omega-squared Fixed-effect	,014	-,006	,035
	Omega-squared Random-effect	,003	-,002	,009

a. Eta-squared and Epsilon-squared are estimated based on the fixed-effect model.

b. Negative but less biased estimates are retained, not rounded to zero.

Post Hoc Tests

Multiple Comparisons

Bonferroni

Dependent Variable	(I) Ethnicitiy in broad categories	(J) Ethnicitiy in broad categories	Mean Difference (I-J)	Std. Error	Sig.	95% Confidence Interval	
						Lower Bound	Upper Bound
Age of subject	The Netherlands	Turkey	7,301*	1,715	<,001	2,47	12,13
		Moroccan	8,803*	1,226	<,001	5,35	12,26
		SE Asian	8,344*	1,351	<,001	4,54	12,15
		Black African	8,612*	1,807	<,001	3,52	13,70
	Turkey	The Netherlands	-7,301*	1,715	<,001	-12,13	-2,47
		Moroccan	1,501	1,937	1,000	-3,96	6,96
		SE Asian	1,043	2,018	1,000	-4,64	6,73
		Black African	1,311	2,348	1,000	-5,30	7,93
	Moroccan	The Netherlands	-8,803*	1,226	<,001	-12,26	-5,35
		Turkey	-1,501	1,937	1,000	-6,96	3,96
		SE Asian	-,458	1,623	1,000	-5,03	4,11
		Black African	-,190	2,019	1,000	-5,88	5,50
	SE Asian	The Netherlands	-8,344*	1,351	<,001	-12,15	-4,54
		Turkey	-1,043	2,018	1,000	-6,73	4,64
		Moroccan	,458	1,623	1,000	-4,11	5,03
		Black African	,268	2,097	1,000	-5,64	6,18
	Black African	The Netherlands	-8,612*	1,807	<,001	-13,70	-3,52
		Turkey	-1,311	2,348	1,000	-7,93	5,30
		Moroccan	,190	2,019	1,000	-5,50	5,88
		SE Asian	-,268	2,097	1,000	-6,18	5,64
Age of diabetes onset	The Netherlands	Turkey	9,04742*	1,69120	<,001	4,2824	13,8124
		Moroccan	9,69275*	1,20898	<,001	6,2864	13,0991
		SE Asian	8,39960*	1,33213	<,001	4,6463	12,1529
		Black African	7,66131*	1,78197	<,001	2,6406	12,6821
	Turkey	The Netherlands	-9,04742*	1,69120	<,001	-13,8124	-4,2824
		Moroccan	,64532	1,90949	1,000	-4,7347	6,0254
		SE Asian	-,64782	1,98975	1,000	-6,2540	4,9583
		Black African	-1,38611	2,31516	1,000	-7,9091	5,1369
	Moroccan	The Netherlands	-9,69275*	1,20898	<,001	-13,0991	-6,2864
		Turkey	-,64532	1,90949	1,000	-6,0254	4,7347
		SE Asian	-1,29314	1,60018	1,000	-5,8017	3,2154
		Black African	-2,03144	1,99033	1,000	-7,6393	3,5764
	SE Asian	The Netherlands	-8,39960*	1,33213	<,001	-12,1529	-4,6463
		Turkey	,64782	1,98975	1,000	-4,9583	6,2540
		Moroccan	1,29314	1,60018	1,000	-3,2154	5,8017
		Black African	-,73829	2,06746	1,000	-6,5634	5,0868
	Black African	The Netherlands	-7,66131*	1,78197	<,001	-12,6821	-2,6406
		Turkey	1,38611	2,31516	1,000	-5,1369	7,9091
		Moroccan	2,03144	1,99033	1,000	-3,5764	7,6393
		SE Asian	,73829	2,06746	1,000	-5,0868	6,5634
Diabetes duration	The Netherlands	Turkey	-1,7460	1,1429	1,000	-4,966	1,474
		Moroccan	-,8901	,8170	1,000	-3,192	1,412
		SE Asian	-,0555	,9003	1,000	-2,592	2,481
		Black African	,9512	1,2043	1,000	-2,442	4,344
	Turkey	The Netherlands	1,7460	1,1429	1,000	-1,474	4,966
		Moroccan	,8559	1,2905	1,000	-2,780	4,492
		SE Asian	1,6906	1,3447	1,000	-2,098	5,479
		Black African	2,6972	1,5646	,852	-1,711	7,106
	Moroccan	The Netherlands	,8901	,8170	1,000	-1,412	3,192
		Turkey	-,8559	1,2905	1,000	-4,492	2,780
		SE Asian	,8347	1,0814	1,000	-2,212	3,882
		Black African	1,8413	1,3451	1,000	-1,948	5,631

	SE Asian	The Netherlands	,0555	,9003	1,000	-2,481	2,592
		Turkey	-1,6906	1,3447	1,000	-5,479	2,098
		Moroccan	-,8347	1,0814	1,000	-3,882	2,212
		Black African	1,0066	1,3972	1,000	-2,930	4,943
	Black African	The Netherlands	-,9512	1,2043	1,000	-4,344	2,442
		Turkey	-2,6972	1,5646	,852	-7,106	1,711
		Moroccan	-1,8413	1,3451	1,000	-5,631	1,948
		SE Asian	-1,0066	1,3972	1,000	-4,943	2,930
Body mass index (kg/m2)	The Netherlands	Turkey	-1,6508	,9016	,676	-4,191	,890
		Moroccan	-,6870	,6386	1,000	-2,486	1,112
		SE Asian	1,8189	,7035	,100	-,163	3,801
		Black African	-,9895	,9513	1,000	-3,670	1,691
	Turkey	The Netherlands	1,6508	,9016	,676	-,890	4,191
		Moroccan	,9638	1,0151	1,000	-1,896	3,824
		SE Asian	3,4697*	1,0572	,011	,491	6,448
		Black African	,6613	1,2360	1,000	-2,821	4,144
	Moroccan	The Netherlands	,6870	,6386	1,000	-1,112	2,486
		Turkey	-,9638	1,0151	1,000	-3,824	1,896
		SE Asian	2,5059*	,8441	,031	,128	4,884
		Black African	-,3025	1,0595	1,000	-3,288	2,683
	SE Asian	The Netherlands	-1,8189	,7035	,100	-3,801	,163
		Turkey	-3,4697*	1,0572	,011	-6,448	-,491
		Moroccan	-2,5059*	,8441	,031	-4,884	-,128
		Black African	-2,8084	1,0998	,109	-5,907	,290
	Black African	The Netherlands	,9895	,9513	1,000	-1,691	3,670
		Turkey	-,6613	1,2360	1,000	-4,144	2,821
		Moroccan	,3025	1,0595	1,000	-2,683	3,288
		SE Asian	2,8084	1,0998	,109	-,290	5,907
hsCRP	The Netherlands	Turkey	-,0899	1,0529	1,000	-3,057	2,877
		Moroccan	-,1467	,7527	1,000	-2,268	1,974
		SE Asian	1,2706	,8294	1,000	-1,066	3,607
		Black African	-,4933	1,1095	1,000	-3,619	2,633
	Turkey	The Netherlands	,0899	1,0529	1,000	-2,877	3,057
		Moroccan	-,0568	1,1889	1,000	-3,406	3,293
		SE Asian	1,3605	1,2388	1,000	-2,130	4,851
		Black African	-,4034	1,4414	1,000	-4,465	3,658
	Moroccan	The Netherlands	,1467	,7527	1,000	-1,974	2,268
		Turkey	,0568	1,1889	1,000	-3,293	3,406
		SE Asian	1,4173	,9963	1,000	-1,390	4,224
		Black African	-,3466	1,2392	1,000	-3,838	3,145
	SE Asian	The Netherlands	-1,2706	,8294	1,000	-3,607	1,066
		Turkey	-1,3605	1,2388	1,000	-4,851	2,130
		Moroccan	-1,4173	,9963	1,000	-4,224	1,390
		Black African	-1,7639	1,2872	1,000	-5,391	1,863
	Black African	The Netherlands	,4933	1,1095	1,000	-2,633	3,619
		Turkey	,4034	1,4414	1,000	-3,658	4,465
		Moroccan	,3466	1,2392	1,000	-3,145	3,838
		SE Asian	1,7639	1,2872	1,000	-1,863	5,391
HbA1c (%)	The Netherlands	Turkey	-1,0983*	,1909	<,001	-1,636	-,560
		Moroccan	-,6971*	,1365	<,001	-1,082	-,313
		SE Asian	-,3504	,1504	,201	-,774	,073
		Black African	-,4633	,2011	,216	-1,030	,103
	Turkey	The Netherlands	1,0983*	,1909	<,001	,560	1,636
		Moroccan	,4012	,2155	,632	-,206	1,008
		SE Asian	,7478*	,2246	,009	,115	1,381
		Black African	,6350	,2613	,154	-,101	1,371
	Moroccan	The Netherlands	,6971*	,1365	<,001	,313	1,082
		Turkey	-,4012	,2155	,632	-1,008	,206
		SE Asian	,3467	,1806	,554	-,162	,856
		Black African	,2338	,2247	1,000	-,399	,867
	SE Asian	The Netherlands	,3504	,1504	,201	-,073	,774

		Turkey	-,7478*	,2246	,009	-1,381	-,115
		Moroccan	-,3467	,1806	,554	-,856	,162
		Black African	-,1128	,2334	1,000	-,770	,545
	Black African	The Netherlands	,4633	,2011	,216	-,103	1,030
		Turkey	-,6350	,2613	,154	-1,371	,101
		Moroccan	-,2338	,2247	1,000	-,867	,399
		SE Asian	,1128	,2334	1,000	-,545	,770
Fasting glucose (mmol/L)	The Netherlands	Turkey	-,3467	,4231	1,000	-1,539	,845
		Moroccan	-,6505	,3025	,319	-1,503	,202
		SE Asian	,0708	,3333	1,000	-,868	1,010
		Black African	,4336	,4458	1,000	-,823	1,690
	Turkey	The Netherlands	,3467	,4231	1,000	-,845	1,539
		Moroccan	-,3038	,4777	1,000	-1,650	1,042
		SE Asian	,4175	,4978	1,000	-,985	1,820
		Black African	,7803	,5792	1,000	-,852	2,412
	Moroccan	The Netherlands	,6505	,3025	,319	-,202	1,503
		Turkey	,3038	,4777	1,000	-1,042	1,650
		SE Asian	,7213	,4003	,721	-,407	1,849
		Black African	1,0841	,4979	,299	-,319	2,487
	SE Asian	The Netherlands	-,0708	,3333	1,000	-1,010	,868
		Turkey	-,4175	,4978	1,000	-1,820	,985
		Moroccan	-,7213	,4003	,721	-1,849	,407
		Black African	,3628	,5172	1,000	-1,095	1,820
	Black African	The Netherlands	-,4336	,4458	1,000	-1,690	,823
		Turkey	-,7803	,5792	1,000	-2,412	,852
		Moroccan	-1,0841	,4979	,299	-2,487	,319
		SE Asian	-,3628	,5172	1,000	-1,820	1,095
Total cholesterol	The Netherlands	Turkey	,1259	,1602	1,000	-,325	,577
		Moroccan	,0499	,1145	1,000	-,273	,373
		SE Asian	,1534	,1262	1,000	-,202	,509
		Black African	-,3568	,1688	,349	-,832	,119
	Turkey	The Netherlands	-,1259	,1602	1,000	-,577	,325
		Moroccan	-,0760	,1808	1,000	-,585	,434
		SE Asian	,0275	,1884	1,000	-,503	,558
		Black African	-,4826	,2193	,281	-1,100	,135
	Moroccan	The Netherlands	-,0499	,1145	1,000	-,373	,273
		Turkey	,0760	,1808	1,000	-,434	,585
		SE Asian	,1034	,1515	1,000	-,324	,530
		Black African	-,4067	,1885	,314	-,938	,124
	SE Asian	The Netherlands	-,1534	,1262	1,000	-,509	,202
		Turkey	-,0275	,1884	1,000	-,558	,503
		Moroccan	-,1034	,1515	1,000	-,530	,324
		Black African	-,5101	,1958	,094	-1,062	,042
	Black African	The Netherlands	,3568	,1688	,349	-,119	,832
		Turkey	,4826	,2193	,281	-,135	1,100
		Moroccan	,4067	,1885	,314	-,124	,938
		SE Asian	,5101	,1958	,094	-,042	1,062
HDL cholesterol	The Netherlands	Turkey	,1528*	,0533	,043	,003	,303
		Moroccan	,0560	,0381	1,000	-,051	,163
		SE Asian	,0253	,0420	1,000	-,093	,144
		Black African	-,2448*	,0561	<,001	-,403	-,087
	Turkey	The Netherlands	-,1528*	,0533	,043	-,303	-,003
		Moroccan	-,0968	,0601	1,000	-,266	,073
		SE Asian	-,1275	,0627	,424	-,304	,049
		Black African	-,3976*	,0729	<,001	-,603	-,192
	Moroccan	The Netherlands	-,0560	,0381	1,000	-,163	,051
		Turkey	,0968	,0601	1,000	-,073	,266
		SE Asian	-,0307	,0504	1,000	-,173	,111
		Black African	-,3008*	,0627	<,001	-,477	-,124
	SE Asian	The Netherlands	-,0253	,0420	1,000	-,144	,093
		Turkey	,1275	,0627	,424	-,049	,304

		Moroccan	,0307	,0504	1,000	-,111	,173
		Black African	-,2701*	,0651	<,001	-,454	-,087
	Black African	The Netherlands	,2448*	,0561	<,001	,087	,403
		Turkey	,3976*	,0729	<,001	,192	,603
		Moroccan	,3008*	,0627	<,001	,124	,477
		SE Asian	,2701*	,0651	<,001	,087	,454
Triglycerides	The Netherlands	Turkey	-,0722	,2774	1,000	-,854	,709
		Moroccan	,3445	,1983	,828	-,214	,903
		SE Asian	,3282	,2185	1,000	-,287	,944
		Black African	,5725	,2922	,506	-,251	1,396
	Turkey	The Netherlands	,0722	,2774	1,000	-,709	,854
		Moroccan	,4167	,3132	1,000	-,466	1,299
		SE Asian	,4004	,3263	1,000	-,519	1,320
		Black African	,6447	,3797	,900	-,425	1,714
	Moroccan	The Netherlands	-,3445	,1983	,828	-,903	,214
		Turkey	-,4167	,3132	1,000	-1,299	,466
		SE Asian	-,0163	,2624	1,000	-,756	,723
		Black African	,2280	,3264	1,000	-,692	1,148
	SE Asian	The Netherlands	-,3282	,2185	1,000	-,944	,287
		Turkey	-,4004	,3263	1,000	-1,320	,519
		Moroccan	,0163	,2624	1,000	-,723	,756
		Black African	,2443	,3391	1,000	-,711	1,200
	Black African	The Netherlands	-,5725	,2922	,506	-1,396	,251
		Turkey	-,6447	,3797	,900	-1,714	,425
		Moroccan	-,2280	,3264	1,000	-1,148	,692
		SE Asian	-,2443	,3391	1,000	-1,200	,711
LDL cholesterol	The Netherlands	Turkey	,0706	,1273	1,000	-,288	,429
		Moroccan	-,1292	,0901	1,000	-,383	,125
		SE Asian	,0030	,0993	1,000	-,277	,283
		Black African	-,4222*	,1343	,018	-,801	-,044
	Turkey	The Netherlands	-,0706	,1273	1,000	-,429	,288
		Moroccan	-,1998	,1434	1,000	-,604	,204
		SE Asian	-,0677	,1493	1,000	-,488	,353
		Black African	-,4929*	,1746	,049	-,985	-,001
	Moroccan	The Netherlands	,1292	,0901	1,000	-,125	,383
		Turkey	,1998	,1434	1,000	-,204	,604
		SE Asian	,1321	,1192	1,000	-,204	,468
		Black African	-,2931	,1497	,507	-,715	,129
	SE Asian	The Netherlands	-,0030	,0993	1,000	-,283	,277
		Turkey	,0677	,1493	1,000	-,353	,488
		Moroccan	-,1321	,1192	1,000	-,468	,204
		Black African	-,4252	,1554	,064	-,863	,013
	Black African	The Netherlands	,4222*	,1343	,018	,044	,801
		Turkey	,4929*	,1746	,049	,001	,985
		Moroccan	,2931	,1497	,507	-,129	,715
		SE Asian	,4252	,1554	,064	-,013	,863

*. The mean difference is significant at the 0.05 level.
