# Supplementary material for: Genetic glucocorticoid receptor variants differ between ethnic groups but do not explain variation in age of diabetes onset, metabolic and inflammation parameters in patients with type 2 diabetes
Source: Front Endocrinol (Lausanne). 2023 Sep 4;14:1200183. doi: 10.3389/fendo.2023.1200183 (PMC10507347; doi:10.3389/fendo.2023.1200183)
Supplement: Supplementary file 3 [file DataSheet_2.pdf]

## SUPPLEMENTARY DATA REGRESSION ANALYSIS TABLE 3

### Regression

**Variables Entered/Removed**[a]

| Model | Variables Entered | Variables Removed | Method |
|---|---|---|---|
| 1 | Y_Tth4, Ethnicitiy in broad categories, Sex[b] | . | Enter |

a. Dependent Variable: Age of diabetes onset

b. All requested variables entered.

**Model Summary**

| Model | R | R Square | Adjusted R Square | Std. Error of the Estimate |
|---|---|---|---|---|
| 1 | ,318[a] | ,101 | ,096 | 10,92397 |

a. Predictors: (Constant), Y_Tth4, Ethnicitiy in broad categories, Sex

**ANOVA**[a]

| Model | | Sum of Squares | df | Mean Square | F | Sig. |
|---|---|---|---|---|---|---|
| 1 | Regression | 7748,009 | 3 | 2582,670 | 21,643 | <,001[b] |
| | Residual | 69093,926 | 579 | 119,333 | | |
| | Total | 76841,935 | 582 | | | |

a. Dependent Variable: Age of diabetes onset

b. Predictors: (Constant), Y_Tth4, Ethnicitiy in broad categories, Sex

**Coefficients**[a]

| Model | | Unstandardized Coefficients | | Standardized Coefficients | |
|---|---|---|---|---|---|
| | | B | Std. Error | Beta | t |
| 1 | (Constant) | 56,493 | 1,626 | | 34,749 |
| | Ethnicitiy in broad categories | -2,700 | ,336 | -,320 | -8,040 |
| | Sex | ,542 | ,916 | ,024 | ,592 |
| | Y_Tth4 | -,516 | ,907 | -,022 | -,568 |

**Coefficients**[a]

| Model | | Sig. |
|---|---|---|
| 1 | (Constant) | <,001 |
| | Ethnicitiy in broad categories | <,001 |
| | Sex | ,554 |
| | Y_Tth4 | ,570 |

a. Dependent Variable: Age of diabetes onset

## Regression

**Variables Entered/Removed**[a]

| Model | Variables Entered | Variables Removed | Method |
|---|---|---|---|
| 1 | Y_ER2223EK, Sex, Ethnicitiy in broad categories[b] | . | Enter |

a. Dependent Variable: Age of diabetes onset

b. All requested variables entered.

**Model Summary**

| Model | R | R Square | Adjusted R Square | Std. Error of the Estimate |
|---|---|---|---|---|
| 1 | ,327[a] | ,107 | ,102 | 10,85586 |

a. Predictors: (Constant), Y_ER2223EK, Sex, Ethnicitiy in broad categories

**ANOVA**[a]

| Model | | Sum of Squares | df | Mean Square | F | Sig. |
|---|---|---|---|---|---|---|
| 1 | Regression | 8321,148 | 3 | 2773,716 | 23,536 | <,001[b] |
| | Residual | 69531,337 | 590 | 117,850 | | |
| | Total | 77852,485 | 593 | | | |

a. Dependent Variable: Age of diabetes onset

b. Predictors: (Constant), Y_ER2223EK, Sex, Ethnicitiy in broad categories

**Coefficients**[a]

| Model | | Unstandardized Coefficients | | Standardized Coefficients | |
|---|---|---|---|---|---|
| | | B | Std. Error | Beta | t |
| 1 | (Constant) | 56,224 | 2,801 | | 20,070 |
| | Ethnicitiy in broad categories | -2,763 | ,331 | -,328 | -8,349 |
| | Sex | ,268 | ,902 | ,012 | ,297 |
| | Y_ER2223EK | -,124 | 2,536 | -,002 | -,049 |

**Coefficients**[a]

| Model | | Sig. |
|---|---|---|
| 1 | (Constant) | <,001 |
| | Ethnicitiy in broad categories | <,001 |
| | Sex | ,767 |
| | Y_ER2223EK | ,961 |

a. Dependent Variable: Age of diabetes onset

## Regression

### Variables Entered/Removed[a]

| Model | Variables Entered | Variables Removed | Method |
|---|---|---|---|
| 1 | Y_N363S, Sex, Ethnicitiy in broad categories[b] | . | Enter |

a. Dependent Variable: Age of diabetes onset

b. All requested variables entered.

### Model Summary

| Model | R | R Square | Adjusted R Square | Std. Error of the Estimate |
|---|---|---|---|---|
| 1 | ,322[a] | ,104 | ,099 | 10,92309 |

a. Predictors: (Constant), Y_N363S, Sex, Ethnicitiy in broad categories

### ANOVA[a]

| Model | | Sum of Squares | df | Mean Square | F | Sig. |
|---|---|---|---|---|---|---|
| 1 | Regression | 8104,668 | 3 | 2701,556 | 22,642 | <,001[b] |
| | Residual | 70037,264 | 587 | 119,314 | | |
| | Total | 78141,932 | 590 | | | |

a. Dependent Variable: Age of diabetes onset

b. Predictors: (Constant), Y_N363S, Sex, Ethnicitiy in broad categories

### Coefficients[a]

| Model | | Unstandardized Coefficients | | Standardized Coefficients | |
|---|---|---|---|---|---|
| | | B | Std. Error | Beta | t |
| 1 | (Constant) | 55,650 | 2,230 | | 24,958 |
| | Ethnicitiy in broad categories | -2,730 | ,338 | -,322 | -8,083 |
| | Sex | ,243 | ,912 | ,011 | ,266 |
| | Y_N363S | ,292 | 1,856 | ,006 | ,157 |

### Coefficients[a]

| Model | | Sig. |
|---|---|---|
| 1 | (Constant) | <,001 |
| | Ethnicitiy in broad categories | <,001 |
| | Sex | ,790 |
| | Y_N363S | ,875 |

a. Dependent Variable: Age of diabetes onset

## Regression

**Variables Entered/Removed[a]**

| Model | Variables Entered | Variables Removed | Method |
|---|---|---|---|
| 1 | Y_BCL, Sex, Ethnicitiy in broad categories[b] | . | Enter |

a. Dependent Variable: Age of diabetes onset

b. All requested variables entered.

**Model Summary**

| Model | R | R Square | Adjusted R Square | Std. Error of the Estimate |
|---|---|---|---|---|
| 1 | ,317[a] | ,101 | ,096 | 10,95821 |

a. Predictors: (Constant), Y_BCL, Sex, Ethnicitiy in broad categories

**ANOVA[a]**

| Model | | Sum of Squares | df | Mean Square | F | Sig. |
|---|---|---|---|---|---|---|
| 1 | Regression | 7516,299 | 3 | 2505,433 | 20,864 | <,001[b] |
| | Residual | 67246,141 | 560 | 120,082 | | |
| | Total | 74762,440 | 563 | | | |

a. Dependent Variable: Age of diabetes onset

b. Predictors: (Constant), Y_BCL, Sex, Ethnicitiy in broad categories

**Coefficients[a]**

| Model | | Unstandardized Coefficients B | Unstandardized Coefficients Std. Error | Standardized Coefficients Beta | t |
|---|---|---|---|---|---|
| 1 | (Constant) | 56,507 | 1,793 | | 31,519 |
| | Ethnicitiy in broad categories | -2,727 | ,347 | -,323 | -7,869 |
| | Sex | ,811 | ,935 | ,035 | ,868 |
| | Y_BCL | -,513 | ,940 | -,022 | -,546 |

**Coefficients[a]**

| Model | | Sig. |
|---|---|---|
| 1 | (Constant) | <,001 |
| | Ethnicitiy in broad categories | <,001 |
| | Sex | ,386 |
| | Y_BCL | ,586 |

a. Dependent Variable: Age of diabetes onset

## Regression

### Variables Entered/Removed[a]

| Model | Variables Entered | Variables Removed | Method |
|---|---|---|---|
| 1 | Y_@9BETA, Sex, Ethnicitiy in broad categories[b] | . | Enter |

a. Dependent Variable: Age of diabetes onset

b. All requested variables entered.

### Model Summary

| Model | R | R Square | Adjusted R Square | Std. Error of the Estimate |
|---|---|---|---|---|
| 1 | ,326[a] | ,106 | ,102 | 10,85032 |

a. Predictors: (Constant), Y_@9BETA, Sex, Ethnicitiy in broad categories

### ANOVA[a]

| Model | | Sum of Squares | df | Mean Square | F | Sig. |
|---|---|---|---|---|---|---|
| 1 | Regression | 8055,051 | 3 | 2685,017 | 22,807 | <,001[b] |
| | Residual | 67812,140 | 576 | 117,729 | | |
| | Total | 75867,191 | 579 | | | |

a. Dependent Variable: Age of diabetes onset

b. Predictors: (Constant), Y_@9BETA, Sex, Ethnicitiy in broad categories

### Coefficients[a]

| Model | | Unstandardized Coefficients B | Unstandardized Coefficients Std. Error | Standardized Coefficients Beta | t |
|---|---|---|---|---|---|
| 1 | (Constant) | 58,689 | 1,587 | | 36,976 |
| | Ethnicitiy in broad categories | -2,704 | ,335 | -,322 | -8,080 |
| | Sex | ,508 | ,912 | ,022 | ,558 |
| | Y_@9BETA | -2,252 | ,995 | -,089 | -2,263 |

### Coefficients[a]

| Model | | Sig. |
|---|---|---|
| 1 | (Constant) | <,001 |
| | Ethnicitiy in broad categories | <,001 |
| | Sex | ,577 |
| | Y_@9BETA | ,024 |

a. Dependent Variable: Age of diabetes onset

## Regression

**Variables Entered/Removed[a]**

| Model | Variables Entered | Variables Removed | Method |
|---|---|---|---|
| 1 | Y_Tth4, Insuline, Ethnicitiy in broad categories, Sex, Use of metformin, Diabetes duration[b] | . | Enter |

a. Dependent Variable: HbA1c (%)

b. All requested variables entered.

**Model Summary**

| Model | R | R Square | Adjusted R Square | Std. Error of the Estimate |
|---|---|---|---|---|
| 1 | ,425[a] | ,181 | ,172 | 1,1438 |

a. Predictors: (Constant), Y_Tth4, Insuline, Ethnicitiy in broad categories, Sex, Use of metformin, Diabetes duration

**ANOVA[a]**

| Model | | Sum of Squares | df | Mean Square | F | Sig. |
|---|---|---|---|---|---|---|
| 1 | Regression | 165,798 | 6 | 27,633 | 21,123 | <,001[b] |
| | Residual | 752,226 | 575 | 1,308 | | |
| | Total | 918,024 | 581 | | | |

a. Dependent Variable: HbA1c (%)

b. Predictors: (Constant), Y_Tth4, Insuline, Ethnicitiy in broad categories, Sex, Use of metformin, Diabetes duration

**Coefficients[a]**

| Model | | Unstandardized Coefficients B | Unstandardized Coefficients Std. Error | Standardized Coefficients Beta | t |
|---|---|---|---|---|---|
| 1 | (Constant) | 7,547 | ,303 | | 24,867 |
| | Ethnicitiy in broad categories | ,164 | ,035 | ,178 | 4,653 |
| | Sex | -,013 | ,097 | -,005 | -,137 |
| | Diabetes duration | ,019 | ,007 | ,106 | 2,542 |
| | Insuline | -,895 | ,118 | -,321 | -7,608 |
| | Use of metformin | ,114 | ,107 | ,041 | 1,064 |
| | Y_Tth4 | ,148 | ,095 | ,059 | 1,559 |

**Coefficients[a]**

| Model | | Sig. |
|---|---|---|
| 1 | (Constant) | <,001 |
| | Ethnicitiy in broad categories | <,001 |
| | Sex | ,891 |
| | Diabetes duration | ,011 |
| | Insuline | <,001 |
| | Use of metformin | ,288 |
| | Y_Tth4 | ,119 |

a. Dependent Variable: HbA1c (%)

## Regression

**Variables Entered/Removed**[a]

| Model | Variables Entered | Variables Removed | Method |
|---|---|---|---|
| 1 | Y_ER2223EK, Use of metformin, Ethnicitiy in broad categories, Diabetes duration, Sex, Insuline[b] | . | Enter |

a. Dependent Variable: HbA1c (%)

b. All requested variables entered.

**Model Summary**

| Model | R | R Square | Adjusted R Square | Std. Error of the Estimate |
|---|---|---|---|---|
| 1 | ,412[a] | ,170 | ,161 | 1,1492 |

a. Predictors: (Constant), Y_ER2223EK, Use of metformin, Ethnicitiy in broad categories, Diabetes duration, Sex, Insuline

**ANOVA**[a]

| Model | | Sum of Squares | df | Mean Square | F | Sig. |
|---|---|---|---|---|---|---|
| 1 | Regression | 158,193 | 6 | 26,365 | 19,965 | <,001[b] |
| | Residual | 773,877 | 586 | 1,321 | | |
| | Total | 932,069 | 592 | | | |

a. Dependent Variable: HbA1c (%)

b. Predictors: (Constant), Y_ER2223EK, Use of metformin, Ethnicitiy in broad categories, Diabetes duration, Sex, Insuline

**Coefficients**[a]

| Model | | Unstandardized Coefficients B | Unstandardized Coefficients Std. Error | Standardized Coefficients Beta | t |
|---|---|---|---|---|---|
| 1 | (Constant) | 7,976 | ,402 | | 19,833 |
| | Ethnicitiy in broad categories | ,155 | ,035 | ,168 | 4,394 |
| | Sex | ,003 | ,096 | ,001 | ,035 |
| | Diabetes duration | ,020 | ,007 | ,111 | 2,668 |
| | Insuline | -,855 | ,117 | -,308 | -7,280 |
| | Use of metformin | ,115 | ,107 | ,041 | 1,070 |
| | Y_ER2223EK | -,248 | ,270 | -,035 | -,918 |

**Coefficients**[a]

| Model | | Sig. |
|---|---|---|
| 1 | (Constant) | <,001 |
| | Ethnicitiy in broad categories | <,001 |
| | Sex | ,972 |
| | Diabetes duration | ,008 |
| | Insuline | <,001 |
| | Use of metformin | ,285 |
| | Y_ER2223EK | ,359 |

a. Dependent Variable: HbA1c (%)

## Regression

**Variables Entered/Removed**[a]

| Model | Variables Entered | Variables Removed | Method |
|---|---|---|---|
| 1 | Y_N363S, Insuline, Sex, Use of metformin, Ethnicitiy in broad categories, Diabetes duration[b] | . | Enter |

a. Dependent Variable: HbA1c (%)

b. All requested variables entered.

**Model Summary**

| Model | R | R Square | Adjusted R Square | Std. Error of the Estimate |
|---|---|---|---|---|
| 1 | ,414[a] | ,172 | ,163 | 1,1463 |

a. Predictors: (Constant), Y_N363S, Insuline, Sex, Use of metformin, Ethnicitiy in broad categories, Diabetes duration

**ANOVA**[a]

| Model | | Sum of Squares | df | Mean Square | F | Sig. |
|---|---|---|---|---|---|---|
| 1 | Regression | 158,694 | 6 | 26,449 | 20,128 | <,001[b] |
| | Residual | 766,066 | 583 | 1,314 | | |
| | Total | 924,759 | 589 | | | |

a. Dependent Variable: HbA1c (%)

b. Predictors: (Constant), Y_N363S, Insuline, Sex, Use of metformin, Ethnicitiy in broad categories, Diabetes duration

**Coefficients**[a]

| Model | | Unstandardized Coefficients | | Standardized Coefficients | |
|---|---|---|---|---|---|
| | | B | Std. Error | Beta | t |
| 1 | (Constant) | 7,746 | ,336 | | 23,083 |
| | Ethnicitiy in broad categories | ,161 | ,036 | ,175 | 4,535 |
| | Sex | ,019 | ,097 | ,007 | ,194 |
| | Diabetes duration | ,019 | ,007 | ,109 | 2,600 |
| | Insuline | -,858 | ,117 | -,310 | -7,350 |
| | Use of metformin | ,093 | ,107 | ,033 | ,865 |
| | Y_N363S | -,013 | ,195 | -,003 | -,067 |

**Coefficients**[a]

| Model | | Sig. |
|---|---|---|
| 1 | (Constant) | <,001 |
| | Ethnicitiy in broad categories | <,001 |
| | Sex | ,846 |
| | Diabetes duration | ,010 |
| | Insuline | <,001 |
| | Use of metformin | ,387 |
| | Y_N363S | ,947 |

a. Dependent Variable: HbA1c (%)

## Regression

**Variables Entered/Removed**[a]

| Model | Variables Entered | Variables Removed | Method |
|---|---|---|---|
| 1 | Y_BCL, Sex, Insuline, Use of metformin, Ethnicitiy in broad categories, Diabetes duration[b] | . | Enter |

a. Dependent Variable: HbA1c (%)

b. All requested variables entered.

**Model Summary**

| Model | R | R Square | Adjusted R Square | Std. Error of the Estimate |
|---|---|---|---|---|
| 1 | ,425[a] | ,181 | ,172 | 1,1242 |

a. Predictors: (Constant), Y_BCL, Sex, Insuline, Use of metformin, Ethnicitiy in broad categories, Diabetes duration

**ANOVA**[a]

| Model | | Sum of Squares | df | Mean Square | F | Sig. |
|---|---|---|---|---|---|---|
| 1 | Regression | 154,972 | 6 | 25,829 | 20,436 | <,001[b] |
| | Residual | 702,709 | 556 | 1,264 | | |
| | Total | 857,681 | 562 | | | |

a. Dependent Variable: HbA1c (%)

b. Predictors: (Constant), Y_BCL, Sex, Insuline, Use of metformin, Ethnicitiy in broad categories, Diabetes duration

**Coefficients**[a]

| Model | | Unstandardized Coefficients | | Standardized Coefficients | |
|---|---|---|---|---|---|
| | | B | Std. Error | Beta | t |
| 1 | (Constant) | 7,694 | ,309 | | 24,906 |
| | Ethnicitiy in broad categories | ,156 | ,036 | ,172 | 4,372 |
| | Sex | ,005 | ,097 | ,002 | ,053 |
| | Diabetes duration | ,019 | ,007 | ,111 | 2,601 |
| | Insuline | -,893 | ,117 | -,327 | -7,634 |
| | Use of metformin | ,119 | ,107 | ,043 | 1,109 |
| | Y_BCL | ,031 | ,097 | ,013 | ,325 |

**Coefficients**[a]

| Model | | Sig. |
|---|---|---|
| 1 | (Constant) | <,001 |
| | Ethnicitiy in broad categories | <,001 |
| | Sex | ,958 |
| | Diabetes duration | ,010 |
| | Insuline | <,001 |
| | Use of metformin | ,268 |
| | Y_BCL | ,745 |

a. Dependent Variable: HbA1c (%)

## Regression

### Variables Entered/Removed[a]

| Model | Variables Entered | Variables Removed | Method |
|---|---|---|---|
| 1 | Y_@9BETA, Use of metformin, Diabetes duration, Ethnicitiy in broad categories, Sex, Insuline[b] | . | Enter |

a. Dependent Variable: HbA1c (%)

b. All requested variables entered.

### Model Summary

| Model | R | R Square | Adjusted R Square | Std. Error of the Estimate |
|---|---|---|---|---|
| 1 | ,427[a] | ,182 | ,174 | 1,1444 |

a. Predictors: (Constant), Y_@9BETA, Use of metformin, Diabetes duration, Ethnicitiy in broad categories, Sex, Insuline

### ANOVA[a]

| Model | | Sum of Squares | df | Mean Square | F | Sig. |
|---|---|---|---|---|---|---|
| 1 | Regression | 167,068 | 6 | 27,845 | 21,260 | <,001[b] |
| | Residual | 749,150 | 572 | 1,310 | | |
| | Total | 916,218 | 578 | | | |

a. Dependent Variable: HbA1c (%)

b. Predictors: (Constant), Y_@9BETA, Use of metformin, Diabetes duration, Ethnicitiy in broad categories, Sex, Insuline

### Coefficients[a]

| Model | | Unstandardized Coefficients | | Standardized Coefficients | |
|---|---|---|---|---|---|
| | | B | Std. Error | Beta | t |
| 1 | (Constant) | 7,583 | ,302 | | 25,137 |
| | Ethnicitiy in broad categories | ,164 | ,035 | ,178 | 4,633 |
| | Sex | -,006 | ,097 | -,003 | -,066 |
| | Diabetes duration | ,018 | ,007 | ,102 | 2,447 |
| | Insuline | -,906 | ,118 | -,324 | -7,662 |
| | Use of metformin | ,112 | ,107 | ,041 | 1,050 |
| | Y_@9BETA | ,157 | ,105 | ,057 | 1,492 |

### Coefficients[a]

| Model | | Sig. |
|---|---|---|
| 1 | (Constant) | <,001 |
| | Ethnicitiy in broad categories | <,001 |
| | Sex | ,948 |
| | Diabetes duration | ,015 |
| | Insuline | <,001 |
| | Use of metformin | ,294 |
| | Y_@9BETA | ,136 |

a. Dependent Variable: HbA1c (%)

**Regression**

**Variables Entered/Removed**[a]

| Model | Variables Entered | Variables Removed | Method |
|---|---|---|---|
| 1 | Y_Tth4, Age of subject, Sex[b] | . | Enter |

a. Dependent Variable: hsCRP

b. All requested variables entered.

**Model Summary**

| Model | R | R Square | Adjusted R Square | Std. Error of the Estimate |
|---|---|---|---|---|
| 1 | ,174[a] | ,030 | ,025 | 6,6299 |

a. Predictors: (Constant), Y_Tth4, Age of subject, Sex

**ANOVA**[a]

| Model | | Sum of Squares | df | Mean Square | F | Sig. |
|---|---|---|---|---|---|---|
| 1 | Regression | 796,900 | 3 | 265,633 | 6,043 | <,001[b] |
| | Residual | 25449,944 | 579 | 43,955 | | |
| | Total | 26246,844 | 582 | | | |

a. Dependent Variable: hsCRP

b. Predictors: (Constant), Y_Tth4, Age of subject, Sex

**Coefficients**[a]

| Model | | Unstandardized Coefficients | | Standardized Coefficients | t | Sig. |
|---|---|---|---|---|---|---|
| | | B | Std. Error | Beta | | |
| 1 | (Constant) | 4,909 | 1,753 | | 2,800 | ,005 |
| | Sex | 2,273 | ,551 | ,169 | 4,125 | <,001 |
| | Age of subject | -,025 | ,024 | -,043 | -1,054 | ,292 |
| | Y_Tth4 | ,081 | ,550 | ,006 | ,148 | ,882 |

a. Dependent Variable: hsCRP

## Regression

### Variables Entered/Removed[a]

| Model | Variables Entered | Variables Removed | Method |
|---|---|---|---|
| 1 | Y_ER2223EK, Age of subject, Sex[b] | . | Enter |

a. Dependent Variable: hsCRP

b. All requested variables entered.

### Model Summary

| Model | R | R Square | Adjusted R Square | Std. Error of the Estimate |
|---|---|---|---|---|
| 1 | ,172[a] | ,030 | ,025 | 6,5710 |

a. Predictors: (Constant), Y_ER2223EK, Age of subject, Sex

### ANOVA[a]

| Model | | Sum of Squares | df | Mean Square | F | Sig. |
|---|---|---|---|---|---|---|
| 1 | Regression | 775,540 | 3 | 258,513 | 5,987 | <,001[b] |
| | Residual | 25474,983 | 590 | 43,178 | | |
| | Total | 26250,523 | 593 | | | |

a. Dependent Variable: hsCRP

b. Predictors: (Constant), Y_ER2223EK, Age of subject, Sex

### Coefficients[a]

| Model | | Unstandardized Coefficients | | Standardized Coefficients | t | Sig. |
|---|---|---|---|---|---|---|
| | | B | Std. Error | Beta | | |
| 1 | (Constant) | 5,594 | 2,181 | | 2,565 | ,011 |
| | Sex | 2,205 | ,541 | ,166 | 4,079 | <,001 |
| | Age of subject | -,024 | ,024 | -,041 | -1,019 | ,309 |
| | Y_ER2223EK | -,616 | 1,533 | -,016 | -,402 | ,688 |

a. Dependent Variable: hsCRP

## Regression

### Variables Entered/Removed[a]

| Model | Variables Entered | Variables Removed | Method |
|---|---|---|---|
| 1 | Y_N363S, Sex, Age of subject[b] | . | Enter |

a. Dependent Variable: hsCRP

b. All requested variables entered.

### Model Summary

| Model | R | R Square | Adjusted R Square | Std. Error of the Estimate |
|---|---|---|---|---|
| 1 | ,181[a] | ,033 | ,028 | 6,5769 |

a. Predictors: (Constant), Y_N363S, Sex, Age of subject

### ANOVA[a]

| Model | | Sum of Squares | df | Mean Square | F | Sig. |
|---|---|---|---|---|---|---|
| 1 | Regression | 858,161 | 3 | 286,054 | 6,613 | <,001[b] |
| | Residual | 25390,949 | 587 | 43,255 | | |
| | Total | 26249,110 | 590 | | | |

a. Dependent Variable: hsCRP

b. Predictors: (Constant), Y_N363S, Sex, Age of subject

### Coefficients[a]

| Model | | Unstandardized Coefficients | | Standardized Coefficients | t | Sig. |
|---|---|---|---|---|---|---|
| | | B | Std. Error | Beta | | |
| 1 | (Constant) | 5,746 | 1,831 | | 3,139 | ,002 |
| | Sex | 2,339 | ,543 | ,175 | 4,305 | <,001 |
| | Age of subject | -,024 | ,024 | -,041 | -,994 | ,320 |
| | Y_N363S | -,813 | 1,110 | -,030 | -,733 | ,464 |

a. Dependent Variable: hsCRP

## Regression

### Variables Entered/Removed[a]

| Model | Variables Entered | Variables Removed | Method |
|---|---|---|---|
| 1 | Y_BCL, Age of subject, Sex[b] | . | Enter |

a. Dependent Variable: hsCRP

b. All requested variables entered.

### Model Summary

| Model | R | R Square | Adjusted R Square | Std. Error of the Estimate |
|---|---|---|---|---|
| 1 | ,178[a] | ,032 | ,026 | 6,4191 |

a. Predictors: (Constant), Y_BCL, Age of subject, Sex

### ANOVA[a]

| Model | | Sum of Squares | df | Mean Square | F | Sig. |
|---|---|---|---|---|---|---|
| 1 | Regression | 753,332 | 3 | 251,111 | 6,094 | <,001[b] |
| | Residual | 23074,647 | 560 | 41,205 | | |
| | Total | 23827,979 | 563 | | | |

a. Dependent Variable: hsCRP

b. Predictors: (Constant), Y_BCL, Age of subject, Sex

### Coefficients[a]

| Model | | Unstandardized Coefficients | | Standardized Coefficients | t | Sig. |
|---|---|---|---|---|---|---|
| | | B | Std. Error | Beta | | |
| 1 | (Constant) | 3,273 | 1,737 | | 1,885 | ,060 |
| | Sex | 2,223 | ,542 | ,171 | 4,101 | <,001 |
| | Age of subject | -,012 | ,024 | -,020 | -,492 | ,623 |
| | Y_BCL | ,555 | ,543 | ,043 | 1,022 | ,307 |

a. Dependent Variable: hsCRP

## Regression

### Variables Entered/Removed[a]

| Model | Variables Entered | Variables Removed | Method |
|---|---|---|---|
| 1 | Y_@9BETA, Sex, Age of subject[b] | . | Enter |

a. Dependent Variable: hsCRP

b. All requested variables entered.

### Model Summary

| Model | R | R Square | Adjusted R Square | Std. Error of the Estimate |
|---|---|---|---|---|
| 1 | ,177[a] | ,031 | ,026 | 6,6399 |

a. Predictors: (Constant), Y_@9BETA, Sex, Age of subject

### ANOVA[a]

| Model | | Sum of Squares | df | Mean Square | F | Sig. |
|---|---|---|---|---|---|---|
| 1 | Regression | 817,689 | 3 | 272,563 | 6,182 | <,001[b] |
| | Residual | 25394,835 | 576 | 44,088 | | |
| | Total | 26212,524 | 579 | | | |

a. Dependent Variable: hsCRP

b. Predictors: (Constant), Y_@9BETA, Sex, Age of subject

### Coefficients[a]

| Model | | Unstandardized Coefficients B | Unstandardized Coefficients Std. Error | Standardized Coefficients Beta | t | Sig. |
|---|---|---|---|---|---|---|
| 1 | (Constant) | 4,558 | 1,764 | | 2,584 | ,010 |
| | Sex | 2,264 | ,553 | ,168 | 4,096 | <,001 |
| | Age of subject | -,026 | ,024 | -,044 | -1,069 | ,286 |
| | Y_@9BETA | ,411 | ,608 | ,028 | ,676 | ,499 |

a. Dependent Variable: hsCRP

**Regression**

**Variables Entered/Removed**[a]

| Model | Variables Entered | Variables Removed | Method |
|---|---|---|---|
| 1 | Y_Tth4, Use of metformin, Age of subject, Use of any lipid lowering agent, Sex[b] | . | Enter |

a. Dependent Variable: Total cholesterol

b. All requested variables entered.

**Model Summary**

| Model | R | R Square | Adjusted R Square | Std. Error of the Estimate |
|---|---|---|---|---|
| 1 | ,241[a] | ,058 | ,050 | ,9827 |

a. Predictors: (Constant), Y_Tth4, Use of metformin, Age of subject, Use of any lipid lowering agent, Sex

**ANOVA**[a]

| Model | | Sum of Squares | df | Mean Square | F | Sig. |
|---|---|---|---|---|---|---|
| 1 | Regression | 34,380 | 5 | 6,876 | 7,121 | <,001[b] |
| | Residual | 556,219 | 576 | ,966 | | |
| | Total | 590,599 | 581 | | | |

a. Dependent Variable: Total cholesterol

b. Predictors: (Constant), Y_Tth4, Use of metformin, Age of subject, Use of any lipid lowering agent, Sex

**Coefficients**[a]

| Model | | Unstandardized Coefficients B | Unstandardized Coefficients Std. Error | Standardized Coefficients Beta | t |
|---|---|---|---|---|---|
| 1 | (Constant) | 3,386 | ,308 | | 11,000 |
| | Sex | ,268 | ,083 | ,133 | 3,246 |
| | Age of subject | -,002 | ,004 | -,020 | -,484 |
| | Use of any lipid lowering agent | ,489 | ,129 | ,154 | 3,782 |
| | Use of metformin | ,200 | ,091 | ,090 | 2,206 |
| | Y_Tth4 | ,033 | ,082 | ,016 | ,406 |

**Coefficients**[a]

| Model | | Sig. |
|---|---|---|
| 1 | (Constant) | <,001 |
| | Sex | ,001 |
| | Age of subject | ,628 |
| | Use of any lipid lowering agent | <,001 |
| | Use of metformin | ,028 |
| | Y_Tth4 | ,685 |

a. Dependent Variable: Total cholesterol

## Regression

**Variables Entered/Removed[a]**

| Model | Variables Entered | Variables Removed | Method |
|---|---|---|---|
| 1 | Y_ER2223EK, Use of metformin, Age of subject, Use of any lipid lowering agent, Sex[b] | . | Enter |

a. Dependent Variable: Total cholesterol

b. All requested variables entered.

**Model Summary**

| Model | R | R Square | Adjusted R Square | Std. Error of the Estimate |
|---|---|---|---|---|
| 1 | ,253[a] | ,064 | ,056 | ,9818 |

a. Predictors: (Constant), Y_ER2223EK, Use of metformin, Age of subject, Use of any lipid lowering agent, Sex

**ANOVA[a]**

| Model | | Sum of Squares | df | Mean Square | F | Sig. |
|---|---|---|---|---|---|---|
| 1 | Regression | 38,677 | 5 | 7,735 | 8,024 | <,001[b] |
| | Residual | 565,882 | 587 | ,964 | | |
| | Total | 604,559 | 592 | | | |

a. Dependent Variable: Total cholesterol

b. Predictors: (Constant), Y_ER2223EK, Use of metformin, Age of subject, Use of any lipid lowering agent, Sex

**Coefficients[a]**

| Model | | Unstandardized Coefficients | | Standardized Coefficients | |
|---|---|---|---|---|---|
| | | B | Std. Error | Beta | t |
| 1 | (Constant) | 3,591 | ,362 | | 9,921 |
| | Sex | ,273 | ,082 | ,135 | 3,337 |
| | Age of subject | -,001 | ,004 | -,015 | -,387 |
| | Use of any lipid lowering agent | ,536 | ,128 | ,168 | 4,180 |
| | Use of metformin | ,199 | ,090 | ,088 | 2,197 |
| | Y_ER2223EK | -,225 | ,230 | -,039 | -,980 |

**Coefficients[a]**

| Model | | Sig. |
|---|---|---|
| 1 | (Constant) | <,001 |
| | Sex | <,001 |
| | Age of subject | ,699 |
| | Use of any lipid lowering agent | <,001 |
| | Use of metformin | ,028 |
| | Y_ER2223EK | ,328 |

a. Dependent Variable: Total cholesterol

**Regression**

**Variables Entered/Removed**[a]

| Model | Variables Entered | Variables Removed | Method |
|---|---|---|---|
| 1 | Y_N363S, Use of any lipid lowering agent, Use of metformin, Age of subject, Sex[b] | . | Enter |

a. Dependent Variable: Total cholesterol

b. All requested variables entered.

**Model Summary**

| Model | R | R Square | Adjusted R Square | Std. Error of the Estimate |
|---|---|---|---|---|
| 1 | ,261[a] | ,068 | ,060 | ,9525 |

a. Predictors: (Constant), Y_N363S, Use of any lipid lowering agent, Use of metformin, Age of subject, Sex

**ANOVA**[a]

| Model | | Sum of Squares | df | Mean Square | F | Sig. |
|---|---|---|---|---|---|---|
| 1 | Regression | 38,876 | 5 | 7,775 | 8,571 | <,001[b] |
| | Residual | 529,787 | 584 | ,907 | | |
| | Total | 568,663 | 589 | | | |

a. Dependent Variable: Total cholesterol

b. Predictors: (Constant), Y_N363S, Use of any lipid lowering agent, Use of metformin, Age of subject, Sex

**Coefficients**[a]

| Model | | Unstandardized Coefficients B | Unstandardized Coefficients Std. Error | Standardized Coefficients Beta | t |
|---|---|---|---|---|---|
| 1 | (Constant) | 3,532 | ,312 | | 11,316 |
| | Sex | ,309 | ,080 | ,157 | 3,871 |
| | Age of subject | -,002 | ,003 | -,020 | -,493 |
| | Use of any lipid lowering agent | ,530 | ,124 | ,171 | 4,265 |
| | Use of metformin | ,153 | ,088 | ,070 | 1,740 |
| | Y_N363S | -,107 | ,161 | -,027 | -,664 |

**Coefficients**[a]

| Model | | Sig. |
|---|---|---|
| 1 | (Constant) | <,001 |
| | Sex | <,001 |
| | Age of subject | ,622 |
| | Use of any lipid lowering agent | <,001 |
| | Use of metformin | ,082 |
| | Y_N363S | ,507 |

a. Dependent Variable: Total cholesterol

## Regression

### Variables Entered/Removed[a]

| Model | Variables Entered | Variables Removed | Method |
|---|---|---|---|
| 1 | Y_BCL, Age of subject, Use of any lipid lowering agent, Use of metformin, Sex[b] | . | Enter |

a. Dependent Variable: Total cholesterol

b. All requested variables entered.

### Model Summary

| Model | R | R Square | Adjusted R Square | Std. Error of the Estimate |
|---|---|---|---|---|
| 1 | ,256[a] | ,065 | ,057 | ,9737 |

a. Predictors: (Constant), Y_BCL, Age of subject, Use of any lipid lowering agent, Use of metformin, Sex

### ANOVA[a]

| Model | | Sum of Squares | df | Mean Square | F | Sig. |
|---|---|---|---|---|---|---|
| 1 | Regression | 36,930 | 5 | 7,386 | 7,791 | <,001[b] |
| | Residual | 528,073 | 557 | ,948 | | |
| | Total | 565,003 | 562 | | | |

a. Dependent Variable: Total cholesterol

b. Predictors: (Constant), Y_BCL, Age of subject, Use of any lipid lowering agent, Use of metformin, Sex

### Coefficients[a]

| Model | | Unstandardized Coefficients | | Standardized Coefficients | |
|---|---|---|---|---|---|
| | | B | Std. Error | Beta | t |
| 1 | (Constant) | 3,356 | ,319 | | 10,530 |
| | Sex | ,267 | ,083 | ,133 | 3,211 |
| | Age of subject | -,001 | ,004 | -,010 | -,253 |
| | Use of any lipid lowering agent | ,547 | ,132 | ,170 | 4,129 |
| | Use of metformin | ,234 | ,091 | ,105 | 2,553 |
| | Y_BCL | -,050 | ,082 | -,025 | -,611 |

### Coefficients[a]

| Model | | Sig. |
|---|---|---|
| 1 | (Constant) | <,001 |
| | Sex | ,001 |
| | Age of subject | ,800 |
| | Use of any lipid lowering agent | <,001 |
| | Use of metformin | ,011 |
| | Y_BCL | ,541 |

a. Dependent Variable: Total cholesterol

## Regression

### Variables Entered/Removed[a]

| Model | Variables Entered | Variables Removed | Method |
|---|---|---|---|
| 1 | Y_@9BETA, Use of metformin, Age of subject, Use of any lipid lowering agent, Sex[b] | . | Enter |

a. Dependent Variable: Total cholesterol

b. All requested variables entered.

### Model Summary

| Model | R | R Square | Adjusted R Square | Std. Error of the Estimate |
|---|---|---|---|---|
| 1 | ,242[a] | ,058 | ,050 | ,9813 |

a. Predictors: (Constant), Y_@9BETA, Use of metformin, Age of subject, Use of any lipid lowering agent, Sex

### ANOVA[a]

| Model | | Sum of Squares | df | Mean Square | F | Sig. |
|---|---|---|---|---|---|---|
| 1 | Regression | 34,212 | 5 | 6,842 | 7,106 | <,001[b] |
| | Residual | 551,776 | 573 | ,963 | | |
| | Total | 585,988 | 578 | | | |

a. Dependent Variable: Total cholesterol

b. Predictors: (Constant), Y_@9BETA, Use of metformin, Age of subject, Use of any lipid lowering agent, Sex

### Coefficients[a]

| Model | | Unstandardized Coefficients | | Standardized Coefficients | |
|---|---|---|---|---|---|
| | | B | Std. Error | Beta | t |
| 1 | (Constant) | 3,417 | ,307 | | 11,143 |
| | Sex | ,267 | ,083 | ,133 | 3,236 |
| | Age of subject | -,002 | ,004 | -,020 | -,492 |
| | Use of any lipid lowering agent | ,492 | ,129 | ,155 | 3,802 |
| | Use of metformin | ,201 | ,091 | ,091 | 2,222 |
| | Y_@9BETA | ,011 | ,090 | ,005 | ,124 |

### Coefficients[a]

| Model | | Sig. |
|---|---|---|
| 1 | (Constant) | <,001 |
| | Sex | ,001 |
| | Age of subject | ,623 |
| | Use of any lipid lowering agent | <,001 |
| | Use of metformin | ,027 |
| | Y_@9BETA | ,901 |

a. Dependent Variable: Total cholesterol

## Regression

**Variables Entered/Removed**[a]

| Model | Variables Entered | Variables Removed | Method |
|---|---|---|---|
| 1 | Y_Tth4, Use of metformin, Age of subject, Use of any lipid lowering agent, Sex[b] | . | Enter |

a. Dependent Variable: Triglycerides

b. All requested variables entered.

**Model Summary**

| Model | R | R Square | Adjusted R Square | Std. Error of the Estimate |
|---|---|---|---|---|
| 1 | ,110[a] | ,012 | ,004 | 1,7697 |

a. Predictors: (Constant), Y_Tth4, Use of metformin, Age of subject, Use of any lipid lowering agent, Sex

**ANOVA**[a]

| Model | | Sum of Squares | df | Mean Square | F | Sig. |
|---|---|---|---|---|---|---|
| 1 | Regression | 22,100 | 5 | 4,420 | 1,411 | ,218[b] |
| | Residual | 1803,883 | 576 | 3,132 | | |
| | Total | 1825,983 | 581 | | | |

a. Dependent Variable: Triglycerides

b. Predictors: (Constant), Y_Tth4, Use of metformin, Age of subject, Use of any lipid lowering agent, Sex

**Coefficients**[a]

| Model | | Unstandardized Coefficients B | Unstandardized Coefficients Std. Error | Standardized Coefficients Beta | t |
|---|---|---|---|---|---|
| 1 | (Constant) | 2,170 | ,554 | | 3,915 |
| | Sex | -,240 | ,149 | -,068 | -1,616 |
| | Age of subject | -,007 | ,006 | -,046 | -1,098 |
| | Use of any lipid lowering agent | -,250 | ,233 | -,045 | -1,073 |
| | Use of metformin | ,180 | ,163 | ,046 | 1,105 |
| | Y_Tth4 | ,165 | ,147 | ,047 | 1,120 |

**Coefficients**[a]

| Model | | Sig. |
|---|---|---|
| 1 | (Constant) | <,001 |
| | Sex | ,107 |
| | Age of subject | ,273 |
| | Use of any lipid lowering agent | ,284 |
| | Use of metformin | ,270 |
| | Y_Tth4 | ,263 |

a. Dependent Variable: Triglycerides

## Regression

### Variables Entered/Removed[a]

| Model | Variables Entered | Variables Removed | Method |
|---|---|---|---|
| 1 | Y_ER2223EK, Use of metformin, Age of subject, Use of any lipid lowering agent, Sex[b] | . | Enter |

a. Dependent Variable: Triglycerides

b. All requested variables entered.

### Model Summary

| Model | R | R Square | Adjusted R Square | Std. Error of the Estimate |
|---|---|---|---|---|
| 1 | ,100[a] | ,010 | ,002 | 1,7585 |

a. Predictors: (Constant), Y_ER2223EK, Use of metformin, Age of subject, Use of any lipid lowering agent, Sex

### ANOVA[a]

| Model | | Sum of Squares | df | Mean Square | F | Sig. |
|---|---|---|---|---|---|---|
| 1 | Regression | 18,322 | 5 | 3,664 | 1,185 | ,315[b] |
| | Residual | 1815,153 | 587 | 3,092 | | |
| | Total | 1833,474 | 592 | | | |

a. Dependent Variable: Triglycerides

b. Predictors: (Constant), Y_ER2223EK, Use of metformin, Age of subject, Use of any lipid lowering agent, Sex

### Coefficients[a]

| Model | | Unstandardized Coefficients | | Standardized Coefficients | |
|---|---|---|---|---|---|
| | | B | Std. Error | Beta | t |
| 1 | (Constant) | 2,644 | ,648 | | 4,079 |
| | Sex | -,218 | ,146 | -,062 | -1,487 |
| | Age of subject | -,007 | ,006 | -,048 | -1,176 |
| | Use of any lipid lowering agent | -,224 | ,230 | -,040 | -,977 |
| | Use of metformin | ,180 | ,162 | ,046 | 1,113 |
| | Y_ER2223EK | -,236 | ,411 | -,024 | -,574 |

### Coefficients[a]

| Model | | Sig. |
|---|---|---|
| 1 | (Constant) | <,001 |
| | Sex | ,138 |
| | Age of subject | ,240 |
| | Use of any lipid lowering agent | ,329 |
| | Use of metformin | ,266 |
| | Y_ER2223EK | ,566 |

a. Dependent Variable: Triglycerides

## Regression

### Variables Entered/Removed[a]

| Model | Variables Entered | Variables Removed | Method |
|---|---|---|---|
| 1 | Y_N363S, Use of any lipid lowering agent, Use of metformin, Age of subject, Sex[b] | . | Enter |

a. Dependent Variable: Triglycerides

b. All requested variables entered.

### Model Summary

| Model | R | R Square | Adjusted R Square | Std. Error of the Estimate |
|---|---|---|---|---|
| 1 | ,114[a] | ,013 | ,005 | 1,0283 |

a. Predictors: (Constant), Y_N363S, Use of any lipid lowering agent, Use of metformin, Age of subject, Sex

### ANOVA[a]

| Model | | Sum of Squares | df | Mean Square | F | Sig. |
|---|---|---|---|---|---|---|
| 1 | Regression | 8,118 | 5 | 1,624 | 1,535 | ,177[b] |
| | Residual | 617,557 | 584 | 1,057 | | |
| | Total | 625,675 | 589 | | | |

a. Dependent Variable: Triglycerides

b. Predictors: (Constant), Y_N363S, Use of any lipid lowering agent, Use of metformin, Age of subject, Sex

### Coefficients[a]

| Model | | Unstandardized Coefficients | | Standardized Coefficients | |
|---|---|---|---|---|---|
| | | B | Std. Error | Beta | t |
| 1 | (Constant) | 2,277 | ,337 | | 6,756 |
| | Sex | -,092 | ,086 | -,045 | -1,072 |
| | Age of subject | -,006 | ,004 | -,071 | -1,712 |
| | Use of any lipid lowering agent | -,166 | ,134 | -,051 | -1,240 |
| | Use of metformin | -,086 | ,095 | -,038 | -,908 |
| | Y_N363S | ,171 | ,174 | ,041 | ,983 |

### Coefficients[a]

| Model | | Sig. |
|---|---|---|
| 1 | (Constant) | <,001 |
| | Sex | ,284 |
| | Age of subject | ,087 |
| | Use of any lipid lowering agent | ,215 |
| | Use of metformin | ,364 |
| | Y_N363S | ,326 |

a. Dependent Variable: Triglycerides

## Regression

**Variables Entered/Removed**[a]

| Model | Variables Entered | Variables Removed | Method |
|---|---|---|---|
| 1 | Y_BCL, Age of subject, Use of any lipid lowering agent, Use of metformin, Sex[b] | . | Enter |

a. Dependent Variable: Triglycerides

b. All requested variables entered.

**Model Summary**

| Model | R | R Square | Adjusted R Square | Std. Error of the Estimate |
|---|---|---|---|---|
| 1 | ,110[a] | ,012 | ,003 | 1,7899 |

a. Predictors: (Constant), Y_BCL, Age of subject, Use of any lipid lowering agent, Use of metformin, Sex

**ANOVA**[a]

| Model | | Sum of Squares | df | Mean Square | F | Sig. |
|---|---|---|---|---|---|---|
| 1 | Regression | 21,732 | 5 | 4,346 | 1,357 | ,239[b] |
| | Residual | 1784,471 | 557 | 3,204 | | |
| | Total | 1806,202 | 562 | | | |

a. Dependent Variable: Triglycerides

b. Predictors: (Constant), Y_BCL, Age of subject, Use of any lipid lowering agent, Use of metformin, Sex

**Coefficients**[a]

| Model | | Unstandardized Coefficients | | Standardized Coefficients | |
|---|---|---|---|---|---|
| | | B | Std. Error | Beta | t |
| 1 | (Constant) | 2,056 | ,586 | | 3,509 |
| | Sex | -,237 | ,153 | -,066 | -1,551 |
| | Age of subject | -,007 | ,007 | -,043 | -1,026 |
| | Use of any lipid lowering agent | -,205 | ,244 | -,036 | -,840 |
| | Use of metformin | ,191 | ,168 | ,048 | 1,137 |
| | Y_BCL | ,181 | ,152 | ,050 | 1,191 |

**Coefficients**[a]

| Model | | Sig. |
|---|---|---|
| 1 | (Constant) | <,001 |
| | Sex | ,122 |
| | Age of subject | ,305 |
| | Use of any lipid lowering agent | ,401 |
| | Use of metformin | ,256 |
| | Y_BCL | ,234 |

a. Dependent Variable: Triglycerides

## Regression

### Variables Entered/Removed[a]

| Model | Variables Entered | Variables Removed | Method |
|---|---|---|---|
| 1 | Y_@9BETA, Use of metformin, Age of subject, Use of any lipid lowering agent, Sex[b] | . | Enter |

a. Dependent Variable: Triglycerides

b. All requested variables entered.

### Model Summary

| Model | R | R Square | Adjusted R Square | Std. Error of the Estimate |
|---|---|---|---|---|
| 1 | ,100[a] | ,010 | ,001 | 1,7763 |

a. Predictors: (Constant), Y_@9BETA, Use of metformin, Age of subject, Use of any lipid lowering agent, Sex

### ANOVA[a]

| Model | | Sum of Squares | df | Mean Square | F | Sig. |
|---|---|---|---|---|---|---|
| 1 | Regression | 18,217 | 5 | 3,643 | 1,155 | ,330[b] |
| | Residual | 1807,958 | 573 | 3,155 | | |
| | Total | 1826,176 | 578 | | | |

a. Dependent Variable: Triglycerides

b. Predictors: (Constant), Y_@9BETA, Use of metformin, Age of subject, Use of any lipid lowering agent, Sex

### Coefficients[a]

| Model | | Unstandardized Coefficients | | Standardized Coefficients | |
|---|---|---|---|---|---|
| | | B | Std. Error | Beta | t |
| 1 | (Constant) | 2,375 | ,555 | | 4,279 |
| | Sex | -,236 | ,150 | -,066 | -1,576 |
| | Age of subject | -,007 | ,007 | -,046 | -1,105 |
| | Use of any lipid lowering agent | -,236 | ,234 | -,042 | -1,007 |
| | Use of metformin | ,182 | ,164 | ,047 | 1,113 |
| | Y_@9BETA | ,021 | ,164 | ,005 | ,128 |

### Coefficients[a]

| Model | | Sig. |
|---|---|---|
| 1 | (Constant) | <,001 |
| | Sex | ,116 |
| | Age of subject | ,270 |
| | Use of any lipid lowering agent | ,315 |
| | Use of metformin | ,266 |
| | Y_@9BETA | ,898 |

a. Dependent Variable: Triglycerides

## Regression

**Variables Entered/Removed[a]**

| Model | Variables Entered | Variables Removed | Method |
|---|---|---|---|
| 1 | Y_Tth4, Use of metformin, Age of subject, Use of any lipid lowering agent, Sex[b] | . | Enter |

a. Dependent Variable: LDL cholesterol

b. All requested variables entered.

**Model Summary**

| Model | R | R Square | Adjusted R Square | Std. Error of the Estimate |
|---|---|---|---|---|
| 1 | ,284[a] | ,081 | ,073 | ,7629 |

a. Predictors: (Constant), Y_Tth4, Use of metformin, Age of subject, Use of any lipid lowering agent, Sex

**ANOVA[a]**

| Model | | Sum of Squares | df | Mean Square | F | Sig. |
|---|---|---|---|---|---|---|
| 1 | Regression | 29,213 | 5 | 5,843 | 10,038 | <,001[b] |
| | Residual | 332,921 | 572 | ,582 | | |
| | Total | 362,134 | 577 | | | |

a. Dependent Variable: LDL cholesterol

b. Predictors: (Constant), Y_Tth4, Use of metformin, Age of subject, Use of any lipid lowering agent, Sex

**Coefficients[a]**

| Model | | Unstandardized Coefficients | | Standardized Coefficients | |
|---|---|---|---|---|---|
| | | B | Std. Error | Beta | t |
| 1 | (Constant) | 1,733 | ,239 | | 7,240 |
| | Sex | ,128 | ,064 | ,081 | 1,994 |
| | Age of subject | -,004 | ,003 | -,062 | -1,558 |
| | Use of any lipid lowering agent | ,560 | ,100 | ,225 | 5,578 |
| | Use of metformin | ,194 | ,071 | ,111 | 2,753 |
| | Y_Tth4 | -,020 | ,064 | -,013 | -,313 |

**Coefficients[a]**

| Model | | Sig. |
|---|---|---|
| 1 | (Constant) | <,001 |
| | Sex | ,047 |
| | Age of subject | ,120 |
| | Use of any lipid lowering agent | <,001 |
| | Use of metformin | ,006 |
| | Y_Tth4 | ,755 |

a. Dependent Variable: LDL cholesterol

## Regression

**Variables Entered/Removed**[a]

| Model | Variables Entered | Variables Removed | Method |
|---|---|---|---|
| 1 | Y_ER2223EK, Use of metformin, Age of subject, Use of any lipid lowering agent, Sex[b] | . | Enter |

a. Dependent Variable: LDL cholesterol

b. All requested variables entered.

**Model Summary**

| Model | R | R Square | Adjusted R Square | Std. Error of the Estimate |
|---|---|---|---|---|
| 1 | ,296[a] | ,088 | ,080 | ,7642 |

a. Predictors: (Constant), Y_ER2223EK, Use of metformin, Age of subject, Use of any lipid lowering agent, Sex

**ANOVA**[a]

| Model | | Sum of Squares | df | Mean Square | F | Sig. |
|---|---|---|---|---|---|---|
| 1 | Regression | 32,715 | 5 | 6,543 | 11,202 | <,001[b] |
| | Residual | 340,515 | 583 | ,584 | | |
| | Total | 373,229 | 588 | | | |

a. Dependent Variable: LDL cholesterol

b. Predictors: (Constant), Y_ER2223EK, Use of metformin, Age of subject, Use of any lipid lowering agent, Sex

**Coefficients**[a]

| Model | | Unstandardized Coefficients | | Standardized Coefficients | |
|---|---|---|---|---|---|
| | | B | Std. Error | Beta | t |
| 1 | (Constant) | 1,865 | ,282 | | 6,603 |
| | Sex | ,127 | ,064 | ,079 | 1,981 |
| | Age of subject | -,004 | ,003 | -,058 | -1,456 |
| | Use of any lipid lowering agent | ,598 | ,100 | ,239 | 5,995 |
| | Use of metformin | ,192 | ,071 | ,108 | 2,714 |
| | Y_ER2223EK | -,211 | ,179 | -,047 | -1,179 |

**Coefficients**[a]

| Model | | Sig. |
|---|---|---|
| 1 | (Constant) | <,001 |
| | Sex | ,048 |
| | Age of subject | ,146 |
| | Use of any lipid lowering agent | <,001 |
| | Use of metformin | ,007 |
| | Y_ER2223EK | ,239 |

a. Dependent Variable: LDL cholesterol

## Regression

### Variables Entered/Removed[a]

| Model | Variables Entered | Variables Removed | Method |
|---|---|---|---|
| 1 | Y_N363S, Use of any lipid lowering agent, Use of metformin, Age of subject, Sex[b] | . | Enter |

a. Dependent Variable: LDL cholesterol

b. All requested variables entered.

### Model Summary

| Model | R | R Square | Adjusted R Square | Std. Error of the Estimate |
|---|---|---|---|---|
| 1 | ,303[a] | ,092 | ,084 | ,7615 |

a. Predictors: (Constant), Y_N363S, Use of any lipid lowering agent, Use of metformin, Age of subject, Sex

### ANOVA[a]

| Model | | Sum of Squares | df | Mean Square | F | Sig. |
|---|---|---|---|---|---|---|
| 1 | Regression | 34,084 | 5 | 6,817 | 11,755 | <,001[b] |
| | Residual | 336,929 | 581 | ,580 | | |
| | Total | 371,012 | 586 | | | |

a. Dependent Variable: LDL cholesterol

b. Predictors: (Constant), Y_N363S, Use of any lipid lowering agent, Use of metformin, Age of subject, Sex

### Coefficients[a]

| Model | | Unstandardized Coefficients | | Standardized Coefficients | |
|---|---|---|---|---|---|
| | | B | Std. Error | Beta | t |
| 1 | (Constant) | 1,922 | ,250 | | 7,680 |
| | Sex | ,143 | ,064 | ,090 | 2,243 |
| | Age of subject | -,004 | ,003 | -,060 | -1,499 |
| | Use of any lipid lowering agent | ,584 | ,099 | ,234 | 5,882 |
| | Use of metformin | ,189 | ,070 | ,107 | 2,689 |
| | Y_N363S | -,238 | ,129 | -,074 | -1,854 |

### Coefficients[a]

| Model | | Sig. |
|---|---|---|
| 1 | (Constant) | <,001 |
| | Sex | ,025 |
| | Age of subject | ,134 |
| | Use of any lipid lowering agent | <,001 |
| | Use of metformin | ,007 |
| | Y_N363S | ,064 |

a. Dependent Variable: LDL cholesterol

## Regression

### Variables Entered/Removed[a]

| Model | Variables Entered | Variables Removed | Method |
|---|---|---|---|
| 1 | Y_BCL, Age of subject, Use of any lipid lowering agent, Use of metformin, Sex[b] | . | Enter |

a. Dependent Variable: LDL cholesterol

b. All requested variables entered.

### Model Summary

| Model | R | R Square | Adjusted R Square | Std. Error of the Estimate |
|---|---|---|---|---|
| 1 | ,307[a] | ,094 | ,086 | ,7556 |

a. Predictors: (Constant), Y_BCL, Age of subject, Use of any lipid lowering agent, Use of metformin, Sex

### ANOVA[a]

| Model | | Sum of Squares | df | Mean Square | F | Sig. |
|---|---|---|---|---|---|---|
| 1 | Regression | 32,745 | 5 | 6,549 | 11,471 | <,001[b] |
| | Residual | 315,714 | 553 | ,571 | | |
| | Total | 348,459 | 558 | | | |

a. Dependent Variable: LDL cholesterol

b. Predictors: (Constant), Y_BCL, Age of subject, Use of any lipid lowering agent, Use of metformin, Sex

### Coefficients[a]

| Model | | Unstandardized Coefficients | | Standardized Coefficients | |
|---|---|---|---|---|---|
| | | B | Std. Error | Beta | t |
| 1 | (Constant) | 1,702 | ,248 | | 6,871 |
| | Sex | ,132 | ,065 | ,084 | 2,042 |
| | Age of subject | -,004 | ,003 | -,054 | -1,333 |
| | Use of any lipid lowering agent | ,611 | ,103 | ,241 | 5,945 |
| | Use of metformin | ,222 | ,071 | ,127 | 3,117 |
| | Y_BCL | -,086 | ,064 | -,054 | -1,339 |

### Coefficients[a]

| Model | | Sig. |
|---|---|---|
| 1 | (Constant) | <,001 |
| | Sex | ,042 |
| | Age of subject | ,183 |
| | Use of any lipid lowering agent | <,001 |
| | Use of metformin | ,002 |
| | Y_BCL | ,181 |

a. Dependent Variable: LDL cholesterol

## Regression

### Variables Entered/Removed[a]

| Model | Variables Entered | Variables Removed | Method |
|---|---|---|---|
| 1 | Y_@9BETA, Use of metformin, Age of subject, Use of any lipid lowering agent, Sex[b] | . | Enter |

a. Dependent Variable: LDL cholesterol

b. All requested variables entered.

### Model Summary

| Model | R | R Square | Adjusted R Square | Std. Error of the Estimate |
|---|---|---|---|---|
| 1 | ,287[a] | ,082 | ,074 | ,7602 |

a. Predictors: (Constant), Y_@9BETA, Use of metformin, Age of subject, Use of any lipid lowering agent, Sex

### ANOVA[a]

| Model | | Sum of Squares | df | Mean Square | F | Sig. |
|---|---|---|---|---|---|---|
| 1 | Regression | 29,415 | 5 | 5,883 | 10,179 | <,001[b] |
| | Residual | 328,849 | 569 | ,578 | | |
| | Total | 358,263 | 574 | | | |

a. Dependent Variable: LDL cholesterol

b. Predictors: (Constant), Y_@9BETA, Use of metformin, Age of subject, Use of any lipid lowering agent, Sex

### Coefficients[a]

| Model | | Unstandardized Coefficients | | Standardized Coefficients | |
|---|---|---|---|---|---|
| | | B | Std. Error | Beta | t |
| 1 | (Constant) | 1,744 | ,238 | | 7,328 |
| | Sex | ,125 | ,064 | ,079 | 1,952 |
| | Age of subject | -,004 | ,003 | -,064 | -1,591 |
| | Use of any lipid lowering agent | ,565 | ,100 | ,228 | 5,628 |
| | Use of metformin | ,197 | ,070 | ,113 | 2,794 |
| | Y_@9BETA | -,033 | ,070 | -,019 | -,468 |

### Coefficients[a]

| Model | | Sig. |
|---|---|---|
| 1 | (Constant) | <,001 |
| | Sex | ,051 |
| | Age of subject | ,112 |
| | Use of any lipid lowering agent | <,001 |
| | Use of metformin | ,005 |
| | Y_@9BETA | ,640 |

a. Dependent Variable: LDL cholesterol
