# Supplementary material for: Genetic glucocorticoid receptor variants differ between ethnic groups but do not explain variation in age of diabetes onset, metabolic and inflammation parameters in patients with type 2 diabetes
Source: Front Endocrinol (Lausanne). 2023 Sep 4;14:1200183. doi: 10.3389/fendo.2023.1200183 (PMC10507347; doi:10.3389/fendo.2023.1200183)
Supplement: Supplementary file 4 [file DataSheet_3.pdf]

SUPPLEMENTARY DATA LINEAR REGERESSION ANALYSIS TABLE 4

Regression

Variables Entered/Removed[a]

Model	Variables Entered	Variables Removed	Method
1	wild type, Sex, Ethnicitiy in broad categories[b]	.	Enter

a. Dependent Variable: Age of diabetes onset

b. All requested variables entered.

Model Summary

Model	R	R Square	Adjusted R Square	Std. Error of the Estimate
1	,326[a]	,106	,102	10,91278

a. Predictors: (Constant), wild type, Sex, Ethnicitiy in broad categories

ANOVA[a]

Model		Sum of Squares	df	Mean Square	F	Sig.
1	Regression	7853,527	3	2617,842	21,982	<,001[b]
	Residual	65975,182	554	119,089		
	Total	73828,710	557			

a. Dependent Variable: Age of diabetes onset

b. Predictors: (Constant), wild type, Sex, Ethnicitiy in broad categories

Coefficients[a]

Model		Unstandardized Coefficients B	Unstandardized Coefficients Std. Error	Standardized Coefficients Beta	t
1	(Constant)	54,394	1,078		50,452
	Sex	,977	,946	,042	1,033
	Ethnicitiy in broad categories	-2,815	,348	-,334	-8,087
	wild type	1,535	,684	,093	2,242

Coefficients[a]

Model		Sig.
1	(Constant)	<,001
	Sex	,302
	Ethnicitiy in broad categories	<,001

a. Dependent Variable: Age of diabetes onset

Regression

Variables Entered/Removed[a]

Model	Variables Entered	Variables Removed	Method
1	BCL, Sex, Ethnicitiy in broad categories[b]	.	Enter

a. Dependent Variable: Age of diabetes onset

b. All requested variables entered.

Model Summary

Model	R	R Square	Adjusted R Square	Std. Error of the Estimate
1	,317[a]	,101	,096	10,93809

a. Predictors: (Constant), BCL, Sex, Ethnicitiy in broad categories

ANOVA[a]

Model		Sum of Squares	df	Mean Square	F	Sig.
1	Regression	7453,276	3	2484,425	20,766	<,001[b]
	Residual	66640,489	557	119,642		
	Total	74093,765	560			

a. Dependent Variable: Age of diabetes onset

b. Predictors: (Constant), BCL, Sex, Ethnicitiy in broad categories

Coefficients[a]

Model		Unstandardized Coefficients		Standardized Coefficients	
		B	Std. Error	Beta	t
1	(Constant)	56,070	,984		56,997
	Sex	,727	,934	,032	,778
	Ethnicitiy in broad categories	-2,703	,343	-,321	-7,882
	BCL	-,801	,812	-,040	-,986

Coefficients[a]

Model		Sig.
1	(Constant)	<,001
	Sex	,437
	Ethnicitiy in broad categories	<,001
	BCL	,324

a. Dependent Variable: Age of diabetes onset

Regression

Variables Entered/Removed[a]

Model	Variables Entered	Variables Removed	Method
1	Tth4 + @9BETA, Ethnicitiy in broad categories, Sex[b]	.	Enter

a. Dependent Variable: Age of diabetes onset

b. All requested variables entered.

Model Summary

Model	R	R Square	Adjusted R Square	Std. Error of the Estimate
1	,326[a]	,106	,102	10,86742

a. Predictors: (Constant), Tth4 + @9BETA, Ethnicitiy in broad categories, Sex

ANOVA[a]

Model		Sum of Squares	df	Mean Square	F	Sig.
1	Regression	8046,622	3	2682,207	22,711	<,001[b]
	Residual	67553,705	572	118,101		
	Total	75600,326	575			

a. Dependent Variable: Age of diabetes onset

b. Predictors: (Constant), Tth4 + @9BETA, Ethnicitiy in broad categories, Sex

Coefficients[a]

Model		Unstandardized Coefficients		Standardized Coefficients	
		B	Std. Error	Beta	t
1	(Constant)	56,296	,927		60,731
	Sex	,600	,917	,026	,654
	Ethnicitiy in broad categories	-2,665	,336	-,316	-7,930
	Tth4 + @9BETA	-2,326	,926	-,100	-2,513

Coefficients[a]

Model		Sig.
1	(Constant)	<,001
	Sex	,513
	Ethnicitiy in broad categories	<,001
	Tth4 + @9BETA	,012

a. Dependent Variable: Age of diabetes onset

Regression

Variables Entered/Removed[a]

Model	Variables Entered	Variables Removed	Method
1	Tth4 + BCL, Sex, Ethnicitiy in broad categories[b]	.	Enter

a. Dependent Variable: Age of diabetes onset

b. All requested variables entered.

Model Summary

Model	R	R Square	Adjusted R Square	Std. Error of the Estimate
1	,315[a]	,099	,094	10,94582

a. Predictors: (Constant), Tth4 + BCL, Sex, Ethnicitiy in broad categories

ANOVA[a]

Model		Sum of Squares	df	Mean Square	F	Sig.
1	Regression	7359,072	3	2453,024	20,474	<,001[b]
	Residual	66734,693	557	119,811		
	Total	74093,765	560			

a. Dependent Variable: Age of diabetes onset

b. Predictors: (Constant), Tth4 + BCL, Sex, Ethnicitiy in broad categories

Coefficients[a]

Model		Unstandardized Coefficients		Standardized Coefficients	
		B	Std. Error	Beta	t
1	(Constant)	55,578	,951		58,456
	Sex	,677	,936	,029	,724
	Ethnicitiy in broad categories	-2,659	,343	-,316	-7,747
	Tth4 + BCL	,452	1,050	,017	,430

Coefficients[a]

Model		Sig.
1	(Constant)	<,001
	Sex	,470
	Ethnicitiy in broad categories	<,001
	Tth4 + BCL	,667

a. Dependent Variable: Age of diabetes onset

Regression

Variables Entered/Removed[a]

Model	Variables Entered	Variables Removed	Method
1	N363S, Sex, Ethnicitiy in broad categories[b]	.	Enter

a. Dependent Variable: Age of diabetes onset

b. All requested variables entered.

Model Summary

Model	R	R Square	Adjusted R Square	Std. Error of the Estimate
1	,322[a]	,104	,099	10,92332

a. Predictors: (Constant), N363S, Sex, Ethnicitiy in broad categories

ANOVA[a]

Model		Sum of Squares	df	Mean Square	F	Sig.
1	Regression	8101,713	3	2700,571	22,633	<,001[b]
	Residual	70040,219	587	119,319		
	Total	78141,932	590			

a. Dependent Variable: Age of diabetes onset

b. Predictors: (Constant), N363S, Sex, Ethnicitiy in broad categories

Coefficients[a]

Model		Unstandardized Coefficients		Standardized Coefficients	
		B	Std. Error	Beta	t
1	(Constant)	55,972	,904		61,949
	Sex	,254	,912	,011	,278
	Ethnicitiy in broad categories	-2,738	,338	-,323	-8,104
	N363S	,004	1,724	,000	,002

Coefficients[a]

Model		Sig.
1	(Constant)	<,001
	Sex	,781
	Ethnicitiy in broad categories	<,001
	N363S	,998

a. Dependent Variable: Age of diabetes onset

Regression

Variables Entered/Removed[a]

Model	Variables Entered	Variables Removed	Method
1	Tth4 + ER2223EK + @9BETA, Sex, Ethnicitiy in broad categories[b]	.	Enter

a. Dependent Variable: Age of diabetes onset

b. All requested variables entered.

Model Summary

Model	R	R Square	Adjusted R Square	Std. Error of the Estimate
1	,324[a]	,105	,101	10,87350

a. Predictors: (Constant), Tth4 + ER2223EK + @9BETA, Sex, Ethnicitiy in broad categories

ANOVA[a]

Model		Sum of Squares	df	Mean Square	F	Sig.
1	Regression	8216,782	3	2738,927	23,166	<,001[b]
	Residual	69875,705	591	118,233		
	Total	78092,487	594			

a. Dependent Variable: Age of diabetes onset

b. Predictors: (Constant), Tth4 + ER2223EK + @9BETA, Sex, Ethnicitiy in broad categories

Coefficients[a]

Model		Unstandardized Coefficients		Standardized Coefficients	
		B	Std. Error	Beta	t
1	(Constant)	56,057	,884		63,401
	Sex	,194	,902	,008	,214
	Ethnicitiy in broad categories	-2,741	,331	-,326	-8,275
	Tth4 + ER2223EK + @9BETA	-,091	2,540	-,001	-,036

Coefficients[a]

Model		Sig.
1	(Constant)	<,001
	Sex	,830
	Ethnicitiy in broad categories	<,001

a. Dependent Variable: Age of diabetes onset

Regression

Variables Entered/Removed[a]

Model	Variables Entered	Variables Removed	Method
1	wild type, Diabetes duration, Use of metformin, Sex, Ethnicitiy in broad categories, Insuline[b]	.	Enter

a. Dependent Variable: HbA1c (%)

b. All requested variables entered.

Model Summary

Model	R	R Square	Adjusted R Square	Std. Error of the Estimate
1	,430[a]	,185	,176	1,1187

a. Predictors: (Constant), wild type, Diabetes duration, Use of metformin, Sex, Ethnicitiy in broad categories, Insuline

ANOVA[a]

Model		Sum of Squares	df	Mean Square	F	Sig.
1	Regression	156,414	6	26,069	20,831	<,001[b]
	Residual	688,290	550	1,251		
	Total	844,704	556			

a. Dependent Variable: HbA1c (%)

b. Predictors: (Constant), wild type, Diabetes duration, Use of metformin, Sex, Ethnicitiy in broad categories, Insuline

Coefficients[a]

Model		Unstandardized Coefficients		Standardized Coefficients	
		B	Std. Error	Beta	t
1	(Constant)	7,832	,271		28,942
	Sex	,007	,098	,003	,070
	Ethnicitiy in broad categories	,162	,036	,180	4,533
	Diabetes duration	,019	,007	,113	2,644
	Insuline	-,896	,118	-,328	-7,620
	Use of metformin	,092	,107	,034	,858
	wild type	-,078	,070	-,044	-1,113

Coefficients[a]

Model		Sig.
1	(Constant)	<,001
	Sex	,944
	Ethnicitiy in broad categories	<,001
	Diabetes duration	,008
	Insuline	<,001
	Use of metformin	,391
	wild type	,266

a. Dependent Variable: HbA1c (%)

Regression

Variables Entered/Removed[a]

Model	Variables Entered	Variables Removed	Method
1	BCL, Diabetes duration, Sex, Use of metformin, Ethnicitiy in broad categories, Insuline[b]	.	Enter

a. Dependent Variable: HbA1c (%)

b. All requested variables entered.

Model Summary

Model	R	R Square	Adjusted R Square	Std. Error of the Estimate
1	,429[a]	,184	,175	1,1188

a. Predictors: (Constant), BCL, Diabetes duration, Sex, Use of metformin, Ethnicitiy in broad categories, Insuline

ANOVA[a]

Model		Sum of Squares	df	Mean Square	F	Sig.
1	Regression	156,001	6	26,000	20,772	<,001[b]
	Residual	692,175	553	1,252		
	Total	848,177	559			

a. Dependent Variable: HbA1c (%)

b. Predictors: (Constant), BCL, Diabetes duration, Sex, Use of metformin, Ethnicitiy in broad categories, Insuline

Coefficients[a]

Model		Unstandardized Coefficients		Standardized Coefficients	
		B	Std. Error	Beta	t
1	(Constant)	7,752	,264		29,360
	Sex	,022	,096	,009	,230
	Ethnicitiy in broad categories	,155	,035	,172	4,400
	Diabetes duration	,019	,007	,112	2,637
	Insuline	-,898	,117	-,330	-7,698
	Use of metformin	,097	,107	,036	,906
	BCL	,023	,083	,011	,274

Coefficients[a]

Model		Sig.
1	(Constant)	<,001
	Sex	,818
	Ethnicitiy in broad categories	<,001
	Diabetes duration	,009
	Insuline	<,001
	Use of metformin	,365
	BCL	,785

a. Dependent Variable: HbA1c (%)

Regression

Variables Entered/Removed[a]

Model	Variables Entered	Variables Removed	Method
1	Tth4 + @9BETA, Use of metformin, Ethnicitiy in broad categories, Diabetes duration, Sex, Insuline[b]	.	Enter

a. Dependent Variable: HbA1c (%)

b. All requested variables entered.

Model Summary

Model	R	R Square	Adjusted R Square	Std. Error of the Estimate
1	,429[a]	,184	,176	1,1221

a. Predictors: (Constant), Tth4 + @9BETA, Use of metformin, Ethnicitiy in broad categories, Diabetes duration, Sex, Insuline

ANOVA[a]

Model		Sum of Squares	df	Mean Square	F	Sig.
1	Regression	161,514	6	26,919	21,379	<,001[b]
	Residual	715,181	568	1,259		
	Total	876,695	574			

a. Dependent Variable: HbA1c (%)

b. Predictors: (Constant), Tth4 + @9BETA, Use of metformin, Ethnicitiy in broad categories, Diabetes duration, Sex,Insuline

Coefficients[a]

Model		Unstandardized Coefficients		Standardized Coefficients	
		B	Std. Error	Beta	t
1	(Constant)	7,752	,262		29,625
	Sex	-,019	,096	-,007	-,194
	Ethnicitiy in broad categories	,151	,035	,166	4,321
	Diabetes duration	,018	,007	,108	2,557
	Insuline	-,903	,117	-,328	-7,738
	Use of metformin	,120	,105	,044	1,146
	Tth4 + @9BETA	,141	,096	,056	1,472

Coefficients[a]

Model		Sig.
1	(Constant)	<,001
	Sex	,846
	Ethnicitiy in broad categories	<,001
	Diabetes duration	,011
	Insuline	<,001
	Use of metformin	,252
	Tth4 + @9BETA	,142

a. Dependent Variable: HbA1c (%)

Regression

Variables Entered/Removed[a]

Model	Variables Entered	Variables Removed	Method
1	Tth4 + BCL, Use of metformin, Ethnicitiy in broad categories, Diabetes duration, Sex, Insuline[b]	.	Enter

a. Dependent Variable: HbA1c (%)

b. All requested variables entered.

Model Summary

Model	R	R Square	Adjusted R Square	Std. Error of the Estimate
1	,429[a]	,184	,175	1,1189

a. Predictors: (Constant), Tth4 + BCL, Use of metformin, Ethnicitiy in broad categories, Diabetes duration, Sex, Insuline

ANOVA[a]

Model		Sum of Squares	df	Mean Square	F	Sig.
1	Regression	155,916	6	25,986	20,759	<,001[b]
	Residual	692,260	553	1,252		
	Total	848,177	559			

a. Dependent Variable: HbA1c (%)

b. Predictors: (Constant), Tth4 + BCL, Use of metformin, Ethnicitiy in broad categories, Diabetes duration, Sex, Insuline

Coefficients[a]

Model		Unstandardized Coefficients		Standardized Coefficients	t
		B	Std. Error	Beta	
1	(Constant)	7,764	,265		29,252
	Sex	,023	,097	,009	,241
	Ethnicitiy in broad categories	,154	,035	,170	4,365
	Diabetes duration	,019	,007	,112	2,618
	Insuline	-,898	,117	-,330	-7,696
	Use of metformin	,098	,107	,036	,920
	Tth4 + BCL	-,009	,108	-,003	-,085

Coefficients[a]

Model		Sig.
1	(Constant)	<,001
	Sex	,809
	Ethnicitiy in broad categories	<,001
	Diabetes duration	,009
	Insuline	<,001
	Use of metformin	,358

a. Dependent Variable: HbA1c (%)

Regression

Variables Entered/Removed[a]

Model	Variables Entered	Variables Removed	Method
1	N363S, Insuline, Sex, Use of metformin, Ethnicitiy in broad categories, Diabetes duration[b]	.	Enter

a. Dependent Variable: HbA1c (%)

b. All requested variables entered.

Model Summary

Model	R	R Square	Adjusted R Square	Std. Error of the Estimate
1	,414[a]	,172	,163	1,1463

a. Predictors: (Constant), N363S, Insuline, Sex, Use of metformin, Ethnicitiy in broad categories, Diabetes duration

ANOVA[a]

Model		Sum of Squares	df	Mean Square	F	Sig.
1	Regression	158,720	6	26,453	20,133	<,001[b]
	Residual	766,039	583	1,314		
	Total	924,759	589			

a. Dependent Variable: HbA1c (%)

b. Predictors: (Constant), N363S, Insuline, Sex, Use of metformin, Ethnicitiy in broad categories, Diabetes duration

Coefficients[a]

Model		Unstandardized Coefficients		Standardized Coefficients	
		B	Std. Error	Beta	t
1	(Constant)	7,734	,264		29,325
	Sex	,020	,097	,008	,203
	Ethnicitiy in broad categories	,161	,036	,174	4,520
	Diabetes duration	,019	,007	,109	2,606
	Insuline	-,858	,117	-,310	-7,340
	Use of metformin	,093	,107	,033	,864
	N363S	-,029	,181	-,006	-,158

Coefficients[a]

Model		Sig.
1	(Constant)	<,001
	Sex	,839
	Ethnicitiy in broad categories	<,001
	Diabetes duration	,009
	Insuline	<,001
	Use of metformin	,388
	N363S	,875

a. Dependent Variable: HbA1c (%)

Regression

Variables Entered/Removed[a]

Model	Variables Entered	Variables Removed	Method
1	Tth4 + ER2223EK + @9BETA, Use of metformin, Ethnicitiy in broad categories, Diabetes duration, Sex, Insuline[b]	.	Enter

a. Dependent Variable: HbA1c (%)

b. All requested variables entered.

Model Summary

Model	R	R Square	Adjusted R Square	Std. Error of the Estimate
1	,412[a]	,170	,161	1,1483

a. Predictors: (Constant), Tth4 + ER2223EK + @9BETA, Use of metformin, Ethnicitiy in broad categories, Diabetes duration, Sex, Insuline

ANOVA[a]

Model		Sum of Squares	df	Mean Square	F	Sig.
1	Regression	158,139	6	26,357	19,988	<,001[b]
	Residual	774,020	587	1,319		
	Total	932,160	593			

a. Dependent Variable: HbA1c (%)

b. Predictors: (Constant), Tth4 + ER2223EK + @9BETA, Use of metformin, Ethnicitiy in broad categories, Diabetes duration, Sex, Insuline

Coefficients[a]

Model		Unstandardized Coefficients B	Unstandardized Coefficients Std. Error	Standardized Coefficients Beta	t
1	(Constant)	7,724	,265		29,142
	Sex	,002	,096	,001	,018
	Ethnicitiy in broad categories	,155	,035	,168	4,415
	Diabetes duration	,020	,007	,112	2,674
	Insuline	-,854	,117	-,308	-7,279
	Use of metformin	,116	,107	,041	1,079
	Tth4 + ER2223EK + @9BETA	-,247	,270	-,035	-,915

Coefficients[a]

1	(Constant)	<,001
	Sex	,985
	Ethnicitiy in broad categories	<,001
	Diabetes duration	,008
	Insuline	<,001
	Use of metformin	,281
	Tth4 + ER2223EK + @9BETA	,360

a. Dependent Variable: HbA1c (%)

Regression

Variables Entered/Removed[a]

Model	Variables Entered	Variables Removed	Method
1	wild type, Age of subject, Sex[b]	.	Enter

a. Dependent Variable: hsCRP

b. All requested variables entered.

Model Summary

Model	R	R Square	Adjusted R Square	Std. Error of the Estimate
1	,174[a]	,030	,025	6,4475

a. Predictors: (Constant), wild type, Age of subject, Sex

ANOVA[a]

Model		Sum of Squares	df	Mean Square	F	Sig.
1	Regression	719,990	3	239,997	5,773	<,001[b]
	Residual	23029,890	554	41,570		
	Total	23749,880	557			

a. Dependent Variable: hsCRP

b. Predictors: (Constant), wild type, Age of subject, Sex

Coefficients[a]

Model		Unstandardized Coefficients		Standardized Coefficients		
		B	Std. Error	Beta	t	Sig.
1	(Constant)	4,350	1,578		2,757	,006
	Sex	2,232	,552	,171	4,046	<,001
	Age of subject	-,013	,024	-,023	-,543	,587
	wild type	-,130	,396	-,014	-,327	,744

a. Dependent Variable: hsCRP

Regression

Variables Entered/Removed[a]

Model	Variables Entered	Variables Removed	Method
1	BCL, Age of subject, Sex[b]	.	Enter

a. Dependent Variable: hsCRP

b. All requested variables entered.

Model Summary

Model	R	R Square	Adjusted R Square	Std. Error of the Estimate
1	,173[a]	,030	,025	6,4350

a. Predictors: (Constant), BCL, Age of subject, Sex

ANOVA[a]

Model		Sum of Squares	df	Mean Square	F	Sig.
1	Regression	711,107	3	237,036	5,724	<,001[b]
	Residual	23065,069	557	41,409		
	Total	23776,176	560			

a. Dependent Variable: hsCRP

b. Predictors: (Constant), BCL, Age of subject, Sex

Coefficients[a]

Model		Unstandardized Coefficients		Standardized Coefficients		
		B	Std. Error	Beta	t	Sig.
1	(Constant)	4,149	1,537		2,700	,007
	Sex	2,233	,545	,171	4,100	<,001
	Age of subject	-,013	,024	-,023	-,544	,587
	BCL	,143	,476	,013	,301	,764

a. Dependent Variable: hsCRP

Regression

Variables Entered/Removed[a]

Model	Variables Entered	Variables Removed	Method
1	Tth4 + @9BETA, Age of subject, Sex[b]	.	Enter

a. Dependent Variable: hsCRP

b. All requested variables entered.

Model Summary

Model	R	R Square	Adjusted R Square	Std. Error of the Estimate
1	,180[a]	,033	,027	6,5809

a. Predictors: (Constant), Tth4 + @9BETA, Age of subject, Sex

ANOVA[a]

Model		Sum of Squares	df	Mean Square	F	Sig.
1	Regression	834,070	3	278,023	6,420	<,001[b]
	Residual	24772,489	572	43,309		
	Total	25606,559	575			

a. Dependent Variable: hsCRP

b. Predictors: (Constant), Tth4 + @9BETA, Age of subject, Sex

Coefficients[a]

Model		Unstandardized Coefficients B	Unstandardized Coefficients Std. Error	Standardized Coefficients Beta	t	Sig.
1	(Constant)	5,010	1,556		3,220	,001
	Sex	2,328	,551	,174	4,226	<,001
	Age of subject	-,026	,024	-,045	-1,097	,273
	Tth4 + @9BETA	,141	,561	,010	,251	,802

a. Dependent Variable: hsCRP

Regression

Variables Entered/Removed[a]

Model	Variables Entered	Variables Removed	Method
1	Tth4 + BCL, Age of subject, Sex[b]	.	Enter

a. Dependent Variable: hsCRP

b. All requested variables entered.

Model Summary

Model	R	R Square	Adjusted R Square	Std. Error of the Estimate
1	,173[a]	,030	,025	6,4351

a. Predictors: (Constant), Tth4 + BCL, Age of subject, Sex

ANOVA[a]

Model		Sum of Squares	df	Mean Square	F	Sig.
1	Regression	710,310	3	236,770	5,718	<,001[b]
	Residual	23065,866	557	41,411		
	Total	23776,176	560			

a. Dependent Variable: hsCRP

b. Predictors: (Constant), Tth4 + BCL, Age of subject, Sex

Coefficients[a]

Model		Unstandardized Coefficients		Standardized Coefficients	t	Sig.
		B	Std. Error	Beta		
1	(Constant)	4,172	1,530		2,726	,007
	Sex	2,230	,545	,171	4,092	<,001
	Age of subject	-,013	,024	-,023	-,544	,586
	Tth4 + BCL	,164	,615	,011	,267	,790

a. Dependent Variable: hsCRP

Regression

Variables Entered/Removed[a]

Model	Variables Entered	Variables Removed	Method
1	N363S, Sex, Age of subject[b]	.	Enter

a. Dependent Variable: hsCRP

b. All requested variables entered.

Model Summary

Model	R	R Square	Adjusted R Square	Std. Error of the Estimate
1	,180[a]	,032	,028	6,5776

a. Predictors: (Constant), N363S, Sex, Age of subject

ANOVA[a]

Model		Sum of Squares	df	Mean Square	F	Sig.
1	Regression	852,403	3	284,134	6,567	<,001[b]
	Residual	25396,707	587	43,265		
	Total	26249,110	590			

a. Dependent Variable: hsCRP

b. Predictors: (Constant), N363S, Sex, Age of subject

Coefficients[a]

Model		Unstandardized Coefficients		Standardized Coefficients	t	Sig.
		B	Std. Error	Beta		
1	(Constant)	4,945	1,514		3,265	,001
	Sex	2,339	,544	,175	4,303	<,001
	Age of subject	-,024	,024	-,041	-1,008	,314
	N363S	-,654	1,030	-,026	-,635	,526

a. Dependent Variable: hsCRP

Regression

Variables Entered/Removed[a]

Model	Variables Entered	Variables Removed	Method
1	Tth4 + ER2223EK + @9BETA, Age of subject, Sex[b]	.	Enter

a. Dependent Variable: hsCRP

b. All requested variables entered.

Model Summary

Model	R	R Square	Adjusted R Square	Std. Error of the Estimate
1	,173[a]	,030	,025	6,5677

a. Predictors: (Constant), Tth4 + ER2223EK + @9BETA, Age of subject, Sex

ANOVA[a]

Model		Sum of Squares	df	Mean Square	F	Sig.
1	Regression	790,990	3	263,663	6,113	<,001[b]
	Residual	25492,265	591	43,134		
	Total	26283,255	594			

a. Dependent Variable: hsCRP

b. Predictors: (Constant), Tth4 + ER2223EK + @9BETA, Age of subject, Sex

Coefficients[a]

Model		Unstandardized Coefficients B	Unstandardized Coefficients Std. Error	Standardized Coefficients Beta	t
1	(Constant)	5,029	1,514		3,321
	Sex	2,220	,540	,167	4,113
	Age of subject	-,025	,024	-,043	-1,056
	Tth4 + ER2223EK + @9BETA	-,621	1,533	-,016	-,405

Coefficients[a]

Model		Sig.
1	(Constant)	<,001
	Sex	<,001
	Age of subject	,292
	Tth4 + ER2223EK + @9BETA	,686

a. Dependent Variable: hsCRP

Regression

Variables Entered/Removed[a]

Model	Variables Entered	Variables Removed	Method
1	wild type, Age of subject, Use of any lipid lowering agent, Use of metformin, Sex[b]	.	Enter

a. Dependent Variable: Total cholesterol

b. All requested variables entered.

Model Summary

Model	R	R Square	Adjusted R Square	Std. Error of the Estimate
1	,265[a]	,070	,062	,9427

a. Predictors: (Constant), wild type, Age of subject, Use of any lipid lowering agent, Use of metformin, Sex

ANOVA[a]

Model		Sum of Squares	df	Mean Square	F	Sig.
1	Regression	36,952	5	7,390	8,315	<,001[b]
	Residual	489,703	551	,889		
	Total	526,655	556			

a. Dependent Variable: Total cholesterol

b. Predictors: (Constant), wild type, Age of subject, Use of any lipid lowering agent, Use of metformin, Sex

Coefficients[a]

Model		Unstandardized Coefficients		Standardized Coefficients	
		B	Std. Error	Beta	t
1	(Constant)	3,288	,290		11,338
	Sex	,294	,081	,151	3,608
	Age of subject	-,001	,004	-,011	-,267
	Use of metformin	,190	,089	,088	2,133
	Use of any lipid lowering agent	,557	,128	,179	4,343
	wild type	,009	,058	,006	,151

Coefficients[a]

Model		Sig.
1	(Constant)	<,001
	Sex	<,001
	Age of subject	,790
	Use of metformin	,033
	Use of any lipid lowering agent	<,001
	wild type	,880

a. Dependent Variable: Total cholesterol

Regression

Variables Entered/Removed[a]

Model	Variables Entered	Variables Removed	Method
1	BCL, Age of subject, Sex, Use of any lipid lowering agent, Use of metformin[b]	.	Enter

a. Dependent Variable: Total cholesterol

b. All requested variables entered.

Model Summary

Model	R	R Square	Adjusted R Square	Std. Error of the Estimate
1	,261[a]	,068	,060	,9487

a. Predictors: (Constant), BCL, Age of subject, Sex, Use of any lipid lowering agent, Use of metformin

ANOVA[a]

Model		Sum of Squares	df	Mean Square	F	Sig.
1	Regression	36,390	5	7,278	8,087	<,001[b]
	Residual	498,600	554	,900		
	Total	534,990	559			

a. Dependent Variable: Total cholesterol

b. Predictors: (Constant), BCL, Age of subject, Sex, Use of any lipid lowering agent, Use of metformin

Coefficients[a]

Model		Unstandardized Coefficients B	Unstandardized Coefficients Std. Error	Standardized Coefficients Beta	t
1	(Constant)	3,261	,286		11,411
	Sex	,286	,081	,146	3,521
	Age of subject	,000	,004	-,003	-,082
	Use of metformin	,190	,090	,088	2,120
	Use of any lipid lowering agent	,558	,129	,178	4,321
	BCL	-,003	,070	-,002	-,042

Coefficients[a]

Model		Sig.
1	(Constant)	<,001
	Sex	<,001
	Age of subject	,935
	Use of metformin	,034
	Use of any lipid lowering agent	<,001
	BCL	,967

a. Dependent Variable: Total cholesterol

Regression

Variables Entered/Removed[a]

Model	Variables Entered	Variables Removed	Method
1	Tth4 + @9BETA, Use of metformin, Use of any lipid lowering agent, Age of subject, Sex[b]	.	Enter

a. Dependent Variable: Total cholesterol

b. All requested variables entered.

Model Summary

Model	R	R Square	Adjusted R Square	Std. Error of the Estimate
1	,242[a]	,059	,050	,9835

a. Predictors: (Constant), Tth4 + @9BETA, Use of metformin, Use of any lipid lowering agent, Age of subject, Sex

ANOVA[a]

Model		Sum of Squares	df	Mean Square	F	Sig.
1	Regression	34,337	5	6,867	7,099	<,001[b]
	Residual	550,415	569	,967		
	Total	584,752	574			

a. Dependent Variable: Total cholesterol

b. Predictors: (Constant), Tth4 + @9BETA, Use of metformin, Use of any lipid lowering agent, Age of subject, Sex

Coefficients[a]

Model		Unstandardized Coefficients B	Unstandardized Coefficients Std. Error	Standardized Coefficients Beta	t
1	(Constant)	3,424	,289		11,867
	Sex	,270	,083	,134	3,242
	Age of subject	-,002	,004	-,022	-,530
	Use of metformin	,202	,091	,091	2,224
	Use of any lipid lowering agent	,506	,132	,157	3,835
	Tth4 + @9BETA	-,009	,084	-,004	-,104

Coefficients[a]

Model		Sig.
1	(Constant)	<,001
	Sex	,001
	Age of subject	,596
	Use of metformin	,027
	Use of any lipid lowering agent	<,001
	Tth4 + @9BETA	,917

a. Dependent Variable: Total cholesterol

Regression

Variables Entered/Removed[a]

Model	Variables Entered	Variables Removed	Method
1	Tth4 + BCL, Use of metformin, Age of subject, Use of any lipid lowering agent, Sex[b]	.	Enter

a. Dependent Variable: Total cholesterol

b. All requested variables entered.

Model Summary

Model	R	R Square	Adjusted R Square	Std. Error of the Estimate
1	,261[a]	,068	,060	,9486

a. Predictors: (Constant), Tth4 + BCL, Use of metformin, Age of subject, Use of any lipid lowering agent, Sex

ANOVA[a]

Model		Sum of Squares	df	Mean Square	F	Sig.
1	Regression	36,431	5	7,286	8,096	<,001[b]
	Residual	498,560	554	,900		
	Total	534,990	559			

a. Dependent Variable: Total cholesterol

b. Predictors: (Constant), Tth4 + BCL, Use of metformin, Age of subject, Use of any lipid lowering agent, Sex

Coefficients[a]

Model		Unstandardized Coefficients B	Unstandardized Coefficients Std. Error	Standardized Coefficients Beta	t
1	(Constant)	3,265	,285		11,440
	Sex	,286	,081	,146	3,527
	Age of subject	,000	,004	-,003	-,084
	Use of metformin	,189	,089	,088	2,119
	Use of any lipid lowering agent	,558	,129	,178	4,321
	Tth4 + BCL	-,020	,091	-,009	-,217

Coefficients[a]

Model		Sig.
1	(Constant)	<,001
	Sex	<,001
	Age of subject	,933
	Use of metformin	,035
	Use of any lipid lowering agent	<,001
	Tth4 + BCL	,828

a. Dependent Variable: Total cholesterol

Regression

Variables Entered/Removed[a]

Model	Variables Entered	Variables Removed	Method
1	N363S, Use of any lipid lowering agent, Use of metformin, Age of subject, Sex[b]	.	Enter

a. Dependent Variable: Total cholesterol

b. All requested variables entered.

Model Summary

Model	R	R Square	Adjusted R Square	Std. Error of the Estimate
1	,261[a]	,068	,060	,9526

a. Predictors: (Constant), N363S, Use of any lipid lowering agent, Use of metformin, Age of subject, Sex

ANOVA[a]

Model		Sum of Squares	df	Mean Square	F	Sig.
1	Regression	38,661	5	7,732	8,520	<,001[b]
	Residual	530,002	584	,908		
	Total	568,663	589			

a. Dependent Variable: Total cholesterol

b. Predictors: (Constant), N363S, Use of any lipid lowering agent, Use of metformin, Age of subject, Sex

Coefficients[a]

Model		Unstandardized Coefficients		Standardized Coefficients	
		B	Std. Error	Beta	t
1	(Constant)	3,429	,274		12,517
	Sex	,308	,080	,156	3,860
	Age of subject	-,002	,003	-,021	-,516
	Use of metformin	,153	,088	,070	1,735
	Use of any lipid lowering agent	,530	,124	,171	4,264
	N363S	-,067	,149	-,018	-,451

Coefficients[a]

Model		Sig.
1	(Constant)	<,001
	Sex	<,001
	Age of subject	,606
	Use of metformin	,083
	Use of any lipid lowering agent	<,001
	N363S	,652

a. Dependent Variable: Total cholesterol

Regression

Variables Entered/Removed[a]

Model	Variables Entered	Variables Removed	Method
1	Tth4 + ER2223EK + @9BETA, Use of metformin, Age of subject, Use of any lipid lowering agent, Sex[b]	.	Enter

a. Dependent Variable: Total cholesterol

b. All requested variables entered.

Model Summary

Model	R	R Square	Adjusted R Square	Std. Error of the Estimate
1	,253[a]	,064	,056	,9810

a. Predictors: (Constant), Tth4 + ER2223EK + @9BETA, Use of metformin, Age of subject, Use of any lipid lowering agent, Sex

ANOVA[a]

Model		Sum of Squares	df	Mean Square	F	Sig.
1	Regression	38,697	5	7,739	8,042	<,001[b]
	Residual	565,889	588	,962		
	Total	604,585	593			

a. Dependent Variable: Total cholesterol

b. Predictors: (Constant), Tth4 + ER2223EK + @9BETA, Use of metformin, Age of subject, Use of any lipid lowering agent, Sex

Coefficients[a]

Model		Unstandardized Coefficients B	Unstandardized Coefficients Std. Error	Standardized Coefficients Beta	t
1	(Constant)	3,368	,282		11,927
	Sex	,273	,082	,135	3,347
	Age of subject	-,001	,004	-,016	-,393
	Use of metformin	,199	,090	,088	2,198
	Use of any lipid lowering agent	,536	,128	,168	4,183
	Tth4 + ER2223EK + @9BETA	-,225	,229	-,039	-,981

Coefficients[a]

Model		Sig.
1	(Constant)	<,001
	Sex	<,001
	Age of subject	,695
	Use of metformin	,028
	Use of any lipid lowering agent	<,001
	Tth4 + ER2223EK + @9BETA	,327

a. Dependent Variable: Total cholesterol

Regression

Variables Entered/Removed[a]

Model	Variables Entered	Variables Removed	Method
1	wild type, Age of subject, Use of any lipid lowering agent, Use of metformin, Sex[b]	.	Enter

a. Dependent Variable: Triglycerides

b. All requested variables entered.

Model Summary

Model	R	R Square	Adjusted R Square	Std. Error of the Estimate
1	,122[a]	,015	,006	1,0303

a. Predictors: (Constant), wild type, Age of subject, Use of any lipid lowering agent, Use of metformin, Sex

ANOVA[a]

Model		Sum of Squares	df	Mean Square	F	Sig.
1	Regression	8,872	5	1,774	1,671	,140[b]
	Residual	584,930	551	1,062		
	Total	593,802	556			

a. Dependent Variable: Triglycerides

b. Predictors: (Constant), wild type, Age of subject, Use of any lipid lowering agent, Use of metformin, Sex

Coefficients[a]

Model		Unstandardized Coefficients		Standardized Coefficients	
		B	Std. Error	Beta	t
1	(Constant)	2,501	,317		7,890
	Sex	-,112	,089	-,054	-1,254
	Age of subject	-,005	,004	-,060	-1,410
	Use of metformin	-,086	,097	-,038	-,889
	Use of any lipid lowering agent	-,156	,140	-,047	-1,113
	wild type	-,108	,063	-,072	-1,695

Coefficients[a]

Model		Sig.
1	(Constant)	<,001
	Sex	,210
	Age of subject	,159
	Use of metformin	,374
	Use of any lipid lowering agent	,266
	wild type	,091

a. Dependent Variable: Triglycerides

Regression

Variables Entered/Removed[a]

Model	Variables Entered	Variables Removed	Method
1	BCL, Age of subject, Sex, Use of any lipid lowering agent, Use of metformin[b]	.	Enter

a. Dependent Variable: Triglycerides

b. All requested variables entered.

Model Summary

Model	R	R Square	Adjusted R Square	Std. Error of the Estimate
1	,101[a]	,010	,001	1,0301

a. Predictors: (Constant), BCL, Age of subject, Sex, Use of any lipid lowering agent, Use of metformin

ANOVA[a]

Model		Sum of Squares	df	Mean Square	F	Sig.
1	Regression	6,078	5	1,216	1,146	,335[b]
	Residual	587,841	554	1,061		
	Total	593,919	559			

a. Dependent Variable: Triglycerides

b. Predictors: (Constant), BCL, Age of subject, Sex, Use of any lipid lowering agent, Use of metformin

Coefficients[a]

Model		Unstandardized Coefficients B	Unstandardized Coefficients Std. Error	Standardized Coefficients Beta	t
1	(Constant)	2,360	,310		7,606
	Sex	-,094	,088	-,045	-1,065
	Age of subject	-,005	,004	-,060	-1,414
	Use of metformin	-,081	,097	-,036	-,833
	Use of any lipid lowering agent	-,151	,140	-,046	-1,077
	BCL	,042	,076	,023	,553

Coefficients[a]

Model		Sig.
1	(Constant)	<,001
	Sex	,288
	Age of subject	,158
	Use of metformin	,405
	Use of any lipid lowering agent	,282
	BCL	,581

a. Dependent Variable: Triglycerides

Regression

Variables Entered/Removed[a]

Model	Variables Entered	Variables Removed	Method
1	Tth4 + @9BETA, Use of metformin, Use of any lipid lowering agent, Age of subject, Sex[b]	.	Enter

a. Dependent Variable: Triglycerides

b. All requested variables entered.

Model Summary

Model	R	R Square	Adjusted R Square	Std. Error of the Estimate
1	,100[a]	,010	,001	1,7814

a. Predictors: (Constant), Tth4 + @9BETA, Use of metformin, Use of any lipid lowering agent, Age of subject, Sex

ANOVA[a]

Model		Sum of Squares	df	Mean Square	F	Sig.
1	Regression	18,167	5	3,633	1,145	,335[b]
	Residual	1805,588	569	3,173		
	Total	1823,755	574			

a. Dependent Variable: Triglycerides

b. Predictors: (Constant), Tth4 + @9BETA, Use of metformin, Use of any lipid lowering agent, Age of subject, Sex

Coefficients[a]

Model		Unstandardized Coefficients B	Unstandardized Coefficients Std. Error	Standardized Coefficients Beta	t
1	(Constant)	2,408	,523		4,608
	Sex	-,234	,151	-,065	-1,550
	Age of subject	-,007	,007	-,047	-1,122
	Use of metformin	,180	,164	,046	1,093
	Use of any lipid lowering agent	-,239	,239	-,042	-1,000
	Tth4 + @9BETA	,040	,152	,011	,265

Coefficients[a]

Model		Sig.
1	(Constant)	<,001
	Sex	,122
	Age of subject	,262
	Use of metformin	,275
	Use of any lipid lowering agent	,318
	Tth4 + @9BETA	,791

a. Dependent Variable: Triglycerides

Regression

Variables Entered/Removed[a]

Model	Variables Entered	Variables Removed	Method
1	Tth4 + BCL, Use of metformin, Age of subject, Use of any lipid lowering agent, Sex[b]	.	Enter

a. Dependent Variable: Triglycerides

b. All requested variables entered.

Model Summary

Model	R	R Square	Adjusted R Square	Std. Error of the Estimate
1	,100[a]	,010	,001	1,0302

a. Predictors: (Constant), Tth4 + BCL, Use of metformin, Age of subject, Use of any lipid lowering agent, Sex

ANOVA[a]

Model		Sum of Squares	df	Mean Square	F	Sig.
1	Regression	5,948	5	1,190	1,121	,348[b]
	Residual	587,971	554	1,061		
	Total	593,919	559			

a. Dependent Variable: Triglycerides

b. Predictors: (Constant), Tth4 + BCL, Use of metformin, Age of subject, Use of any lipid lowering agent, Sex

Coefficients[a]

Model		Unstandardized Coefficients		Standardized Coefficients	
		B	Std. Error	Beta	t
1	(Constant)	2,366	,310		7,633
	Sex	-,095	,088	-,046	-1,073
	Age of subject	-,005	,004	-,060	-1,415
	Use of metformin	-,077	,097	-,034	-,798
	Use of any lipid lowering agent	-,152	,140	-,046	-1,088
	Tth4 + BCL	,042	,098	,018	,427

Coefficients[a]

Model		Sig.
1	(Constant)	<,001
	Sex	,284
	Age of subject	,158
	Use of metformin	,425
	Use of any lipid lowering agent	,277
	Tth4 + BCL	,669

a. Dependent Variable: Triglycerides

Regression

Variables Entered/Removed[a]

Model	Variables Entered	Variables Removed	Method
1	N363S, Use of any lipid lowering agent, Use of metformin, Age of subject, Sex[b]	.	Enter

a. Dependent Variable: Triglycerides

b. All requested variables entered.

Model Summary

Model	R	R Square	Adjusted R Square	Std. Error of the Estimate
1	,112[a]	,013	,004	1,0285

a. Predictors: (Constant), N363S, Use of any lipid lowering agent, Use of metformin, Age of subject, Sex

ANOVA[a]

Model		Sum of Squares	df	Mean Square	F	Sig.
1	Regression	7,903	5	1,581	1,494	,190[b]
	Residual	617,771	584	1,058		
	Total	625,675	589			

a. Dependent Variable: Triglycerides

b. Predictors: (Constant), N363S, Use of any lipid lowering agent, Use of metformin, Age of subject, Sex

Coefficients[a]

Model		Unstandardized Coefficients		Standardized Coefficients	
		B	Std. Error	Beta	t
1	(Constant)	2,444	,296		8,264
	Sex	-,093	,086	-,045	-1,074
	Age of subject	-,006	,004	-,070	-1,697
	Use of metformin	-,086	,095	-,037	-,901
	Use of any lipid lowering agent	-,166	,134	-,051	-1,237
	N363S	,141	,161	,036	,873

Coefficients[a]

Model		Sig.
1	(Constant)	<,001
	Sex	,283
	Age of subject	,090
	Use of metformin	,368
	Use of any lipid lowering agent	,217
	N363S	,383

a. Dependent Variable: Triglycerides

Regression

Variables Entered/Removed[a]

Model	Variables Entered	Variables Removed	Method
1	Tth4 + ER2223EK + @9BETA, Use of metformin, Age of subject, Use of any lipid lowering agent, Sex[b]	.	Enter

a. Dependent Variable: Triglycerides

b. All requested variables entered.

Model Summary

Model	R	R Square	Adjusted R Square	Std. Error of the Estimate
1	,100[a]	,010	,002	1,7577

a. Predictors: (Constant), Tth4 + ER2223EK + @9BETA, Use of metformin, Age of subject, Use of any lipid lowering agent, Sex

ANOVA[a]

Model		Sum of Squares	df	Mean Square	F	Sig.
1	Regression	18,324	5	3,665	1,186	,314[b]
	Residual	1816,596	588	3,089		
	Total	1834,920	593			

a. Dependent Variable: Triglycerides

b. Predictors: (Constant), Tth4 + ER2223EK + @9BETA, Use of metformin, Age of subject, Use of any lipid lowering agent, Sex

Coefficients[a]

Model		Unstandardized Coefficients		Standardized Coefficients	
		B	Std. Error	Beta	t
1	(Constant)	2,388	,506		4,721
	Sex	-,222	,146	-,063	-1,521
	Age of subject	-,007	,006	-,047	-1,141
	Use of metformin	,182	,162	,047	1,126
	Use of any lipid lowering agent	-,222	,229	-,040	-,965
	Tth4 + ER2223EK + @9BETA	-,235	,411	-,024	-,572

Coefficients[a]

Model		Sig.
1	(Constant)	<,001
	Sex	,129
	Age of subject	,254
	Use of metformin	,261
	Use of any lipid lowering agent	,335
	Tth4 + ER2223EK + @9BETA	,568

a. Dependent Variable: Triglycerides

Regression

Variables Entered/Removed[a]

Model	Variables Entered	Variables Removed	Method
1	wild type, Age of subject, Use of any lipid lowering agent, Use of metformin, Sex[b]	.	Enter

a. Dependent Variable: LDL cholesterol

b. All requested variables entered.

Model Summary

Model	R	R Square	Adjusted R Square	Std. Error of the Estimate
1	,310[a]	,096	,088	,7497

a. Predictors: (Constant), wild type, Age of subject, Use of any lipid lowering agent, Use of metformin, Sex

ANOVA[a]

Model		Sum of Squares	df	Mean Square	F	Sig.
1	Regression	32,857	5	6,571	11,692	<,001[b]
	Residual	307,997	548	,562		
	Total	340,854	553			

a. Dependent Variable: LDL cholesterol

b. Predictors: (Constant), wild type, Age of subject, Use of any lipid lowering agent, Use of metformin, Sex

Coefficients[a]

Model		Unstandardized Coefficients		Standardized Coefficients	
		B	Std. Error	Beta	t
1	(Constant)	1,519	,232		6,558
	Sex	,143	,065	,091	2,194
	Age of subject	-,004	,003	-,058	-1,429
	Use of metformin	,224	,071	,130	3,169
	Use of any lipid lowering agent	,617	,102	,246	6,041
	wild type	,055	,046	,049	1,187

Coefficients[a]

Model		Sig.
1	(Constant)	<,001
	Sex	,029
	Age of subject	,154
	Use of metformin	,002
	Use of any lipid lowering agent	<,001
	wild type	,236

a. Dependent Variable: LDL cholesterol

Regression

Variables Entered/Removed[a]

Model	Variables Entered	Variables Removed	Method
1	BCL, Age of subject, Sex, Use of any lipid lowering agent, Use of metformin[b]	.	Enter

a. Dependent Variable: LDL cholesterol

b. All requested variables entered.

Model Summary

Model	R	R Square	Adjusted R Square	Std. Error of the Estimate
1	,301[a]	,091	,082	,7569

a. Predictors: (Constant), BCL, Age of subject, Sex, Use of any lipid lowering agent, Use of metformin

ANOVA[a]

Model		Sum of Squares	df	Mean Square	F	Sig.
1	Regression	31,487	5	6,297	10,993	<,001[b]
	Residual	315,644	551	,573		
	Total	347,131	556			

a. Dependent Variable: LDL cholesterol

b. Predictors: (Constant), BCL, Age of subject, Sex, Use of any lipid lowering agent, Use of metformin

Coefficients[a]

Model		Unstandardized Coefficients		Standardized Coefficients	
		B	Std. Error	Beta	t
1	(Constant)	1,554	,229		6,792
	Sex	,129	,065	,081	1,986
	Age of subject	-,003	,003	-,049	-1,202
	Use of metformin	,220	,071	,126	3,082
	Use of any lipid lowering agent	,614	,103	,243	5,958
	BCL	-,012	,056	-,008	-,206

Coefficients[a]

Model		Sig.
1	(Constant)	<,001
	Sex	,048
	Age of subject	,230
	Use of metformin	,002
	Use of any lipid lowering agent	<,001
	BCL	,837

a. Dependent Variable: LDL cholesterol

Regression

Variables Entered/Removed[a]

Model	Variables Entered	Variables Removed	Method
1	Tth4 + @9BETA, Use of metformin, Use of any lipid lowering agent, Age of subject, Sex[b]	.	Enter

a. Dependent Variable: LDL cholesterol

b. All requested variables entered.

Model Summary

Model	R	R Square	Adjusted R Square	Std. Error of the Estimate
1	,290[a]	,084	,076	,7610

a. Predictors: (Constant), Tth4 + @9BETA, Use of metformin, Use of any lipid lowering agent, Age of subject, Sex

ANOVA[a]

Model		Sum of Squares	df	Mean Square	F	Sig.
1	Regression	29,938	5	5,988	10,339	<,001[b]
	Residual	327,201	565	,579		
	Total	357,139	570			

a. Dependent Variable: LDL cholesterol

b. Predictors: (Constant), Tth4 + @9BETA, Use of metformin, Use of any lipid lowering agent, Age of subject, Sex

Coefficients[a]

Model		Unstandardized Coefficients B	Unstandardized Coefficients Std. Error	Standardized Coefficients Beta	t
1	(Constant)	1,703	,224		7,605
	Sex	,130	,065	,082	2,010
	Age of subject	-,005	,003	-,066	-1,646
	Use of metformin	,196	,071	,113	2,781
	Use of any lipid lowering agent	,579	,102	,229	5,667
	Tth4 + @9BETA	-,030	,065	-,019	-,461

Coefficients[a]

Model		Sig.
1	(Constant)	<,001
	Sex	,045
	Age of subject	,100
	Use of metformin	,006
	Use of any lipid lowering agent	<,001
	Tth4 + @9BETA	,645

a. Dependent Variable: LDL cholesterol

Regression

Variables Entered/Removed[a]

Model	Variables Entered	Variables Removed	Method
1	Tth4 + BCL, Use of metformin, Age of subject, Use of any lipid lowering agent, Sex[b]	.	Enter

a. Dependent Variable: LDL cholesterol

b. All requested variables entered.

Model Summary

Model	R	R Square	Adjusted R Square	Std. Error of the Estimate
1	,302[a]	,091	,083	,7566

a. Predictors: (Constant), Tth4 + BCL, Use of metformin, Age of subject, Use of any lipid lowering agent, Sex

ANOVA[a]

Model		Sum of Squares	df	Mean Square	F	Sig.
1	Regression	31,740	5	6,348	11,090	<,001[b]
	Residual	315,391	551	,572		
	Total	347,131	556			

a. Dependent Variable: LDL cholesterol

b. Predictors: (Constant), Tth4 + BCL, Use of metformin, Age of subject, Use of any lipid lowering agent, Sex

Coefficients[a]

Model		Unstandardized Coefficients B	Unstandardized Coefficients Std. Error	Standardized Coefficients Beta	t
1	(Constant)	1,562	,228		6,843
	Sex	,131	,065	,083	2,013
	Age of subject	-,003	,003	-,049	-1,204
	Use of metformin	,219	,071	,126	3,070
	Use of any lipid lowering agent	,614	,103	,243	5,963
	Tth4 + BCL	-,051	,073	-,028	-,696

Coefficients[a]

Model		Sig.
1	(Constant)	<,001
	Sex	,045
	Age of subject	,229
	Use of metformin	,002
	Use of any lipid lowering agent	<,001
	Tth4 + BCL	,487

a. Dependent Variable: LDL cholesterol

Regression

Variables Entered/Removed[a]

Model	Variables Entered	Variables Removed	Method
1	N363S, Use of metformin, Use of any lipid lowering agent, Age of subject, Sex[b]	.	Enter

a. Dependent Variable: LDL cholesterol

b. All requested variables entered.

Model Summary

Model	R	R Square	Adjusted R Square	Std. Error of the Estimate
1	,300[a]	,090	,082	,7622

a. Predictors: (Constant), N363S, Use of metformin, Use of any lipid lowering agent, Age of subject, Sex

ANOVA[a]

Model		Sum of Squares	df	Mean Square	F	Sig.
1	Regression	33,443	5	6,689	11,512	<,001[b]
	Residual	337,569	581	,581		
	Total	371,012	586			

a. Dependent Variable: LDL cholesterol

b. Predictors: (Constant), N363S, Use of metformin, Use of any lipid lowering agent, Age of subject, Sex

Coefficients[a]

Model		Unstandardized Coefficients B	Unstandardized Coefficients Std. Error	Standardized Coefficients Beta	t
1	(Constant)	1,689	,220		7,682
	Sex	,143	,064	,090	2,237
	Age of subject	-,004	,003	-,061	-1,539
	Use of metformin	,189	,071	,107	2,673
	Use of any lipid lowering agent	,584	,099	,234	5,872
	N363S	-,182	,119	-,061	-1,526

Coefficients[a]

Model		Sig.
1	(Constant)	<,001
	Sex	,026
	Age of subject	,124
	Use of metformin	,008
	Use of any lipid lowering agent	<,001
	N363S	,128

a. Dependent Variable: LDL cholesterol

Regression

Variables Entered/Removed[a]

Model	Variables Entered	Variables Removed	Method
1	Tth4 + ER2223EK + @9BETA, Use of metformin, Age of subject, Use of any lipid lowering agent, Sex[b]	.	Enter

a. Dependent Variable: LDL cholesterol

b. All requested variables entered.

Model Summary

Model	R	R Square	Adjusted R Square	Std. Error of the Estimate
1	,296[a]	,088	,080	,7638

a. Predictors: (Constant), Tth4 + ER2223EK + @9BETA, Use of metformin, Age of subject, Use of any lipid lowering agent, Sex

ANOVA[a]

Model		Sum of Squares	df	Mean Square	F	Sig.
1	Regression	32,745	5	6,549	11,227	<,001[b]
	Residual	340,657	584	,583		
	Total	373,402	589			

a. Dependent Variable: LDL cholesterol

b. Predictors: (Constant), Tth4 + ER2223EK + @9BETA, Use of metformin, Age of subject, Use of any lipid lowering agent, Sex

Coefficients[a]

Model		Unstandardized Coefficients		Standardized Coefficients	
		B	Std. Error	Beta	t
1	(Constant)	1,661	,221		7,530
	Sex	,128	,064	,080	2,007
	Age of subject	-,004	,003	-,059	-1,486
	Use of metformin	,191	,071	,108	2,707
	Use of any lipid lowering agent	,598	,100	,238	5,990
	Tth4 + ER2223EK + @9BETA	-,211	,179	-,047	-1,181

Coefficients[a]

Model		Sig.
1	(Constant)	<,001
	Sex	,045
	Age of subject	,138
	Use of metformin	,007
	Use of any lipid lowering agent	<,001
	Tth4 + ER2223EK + @9BETA	,238

a. Dependent Variable: LDL cholesterol
